# Active coacervate droplets are protocells that grow and resist Ostwald ripening

**DOI:** 10.1038/s41467-021-24111-x

**Published:** 2021-06-21

**Authors:** Karina K. Nakashima, Merlijn H. I. van Haren, Alain A. M. André, Irina Robu, Evan Spruijt

**Affiliations:** Institute for Molecules and Materials, Radboud University, Nijmegen, the Netherlands

**Keywords:** Origin of life, Reaction kinetics and dynamics, Self-assembly

## Abstract

Active coacervate droplets are liquid condensates coupled to a chemical reaction that turns over their components, keeping the droplets out of equilibrium. This turnover can be used to drive active processes such as growth, and provide an insight into the chemical requirements underlying (proto)cellular behaviour. Moreover, controlled growth is a key requirement to achieve population fitness and survival. Here we present a minimal, nucleotide-based coacervate model for active droplets, and report three key findings that make these droplets into evolvable protocells. First, we show that coacervate droplets form and grow by the fuel-driven synthesis of new coacervate material. Second, we find that these droplets do not undergo Ostwald ripening, which we attribute to the attractive electrostatic interactions and translational entropy within complex coacervates, active or passive. Finally, we show that the droplet growth rate reflects experimental conditions such as substrate, enzyme and protein concentration, and that a different droplet composition (addition of RNA) leads to altered growth rates and droplet fitness. These findings together make active coacervate droplets a powerful platform to mimic cellular growth at a single-droplet level, and to study fitness at a population level.

## Abstract (web summary)

Active coacervate droplets are droplets coupled to a chemical reaction that maintains them out of equilibrium, which can be used to drive active processes, but coacervates are still subject to passive processes that compete with or mask growth. Here, the authors present a nucleotide-based model for active coacervate droplets that form and grow by fuel-driven synthesis of ATP, and, importantly, do not undergo Ostwald ripening.

## Introduction

Growth and division are essential processes in life, without which we cannot explain survival and reproduction. Modern cells rely on tightly coordinated mechanisms involving complex machinery, but the sustenance of life-like systems, from their origins to the emergence of a common ancestor, implies that primitive cells lacking similar specialized enzymes could already survive and perhaps even proliferate. This suggests that the behaviour can be reproduced (and explained) using solely chemical principles^1,2^. Such principles may shed light on the emergence of the first cells and help broadening the scope of chemical models used to mimic and decipher biological behaviour^3^. One of the simplest systems predicted to exhibit growth and division is a droplet coupled to a constant supply of droplet material or a chemical reaction: by keeping the reaction out of equilibrium (e.g., with supplied droplet material or fuel for the chemical reaction), the droplet can sustain an active behaviour like growth (i.e., an active droplet)^4–9^. To ensure that the reaction can directly influence behaviour, the droplet must be an open compartment able to exchange material with its surroundings, and compatible with volume change. Coacervates are a promising system to fulfill these requirements^10,11^.

Coacervate droplets form spontaneously by phase separation in a saturated solution of macromolecules; when the phase separation is driven by attractive electrostatic interactions, they are called complex coacervates. Coacervates lack a membrane and thus have no physical barrier that limits their growth. The droplets are permeable to molecules from the surroundings with some selectivity, and concentrate the solutes through dynamic interactions, opening the way for their building blocks to be synthesized in situ^12^. As coacervate droplets are formed by liquid–liquid phase separation, they are in principle governed by the equilibrium concentrations of the building blocks, and when more material is supplied, the volume of the coacervate phase can grow while the overall internal concentration remains approximately constant. This perfectly aligns with the active droplet requirements and is crucial given that most protocell models so far have increased in size via passive mechanisms: vesicle fusion^13^, droplet coalescence and ripening^14,15^, or uptake of externally added building blocks^16^.

**Fig. 1:**
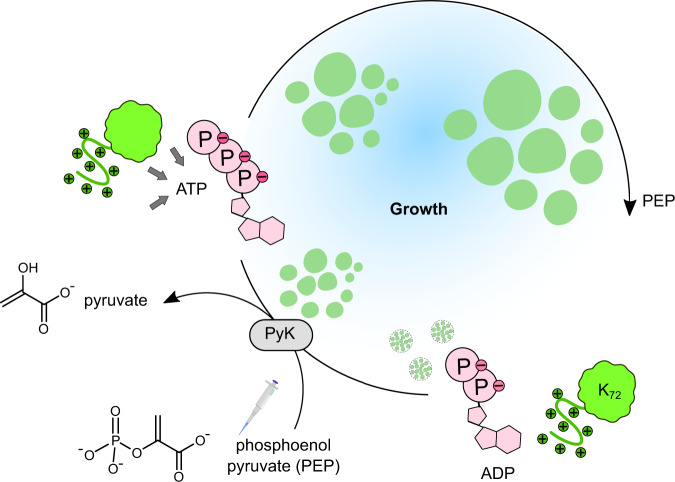
Active droplets scheme. The pyruvate kinase-catalyzed (PyK) conversion of ADP to ATP, combined with the liquid–liquid phase separation of ATP-K_72_ complexes, is a minimal translation of an active droplet. In this system, ADP is the substrate, and ATP (together with the lysine-rich protein K_72_) is the droplet material. We fuel the droplets by manual addition of the second substrate, PEP. The waste, pyruvate, is not re-used in our setup. The local increase in the amount of ATP inside the droplets causes the recruitment of more protein, leading to droplet growth. Growth may compete with other active (nucleation) and passive (coalescence, Ostwald ripening) processes that need to be distinguished experimentally.

Coacervates can achieve growth more easily than vesicles but are still subject to passive processes. Brownian motion-induced coalescence and Ostwald ripening can compete with, or mask, reaction–diffusion-limited growth^17^, and although these processes also lead to an increase in average droplet volume, this comes at the expense of a decreased droplet count—completely disconnected from biological growth. Therefore, for coacervates to hold any potential as dynamic biomimetic models, it is crucial to develop a stable, active system. In addition, growing coacervates must be studied quantitatively and at a single-droplet level in order to undoubtedly distinguish active growth (which we refer to hereafter simply as growth) from passive coarsening. We thus set out to develop an active coacervate model, i.e., one that grows like cells do in two senses: via an increase in droplet volume while keeping droplet count constant (growth), or via an increase in droplet count (nucleation)^18^. Our experimental model for active droplets is based on the pyruvate kinase-catalyzed conversion of ADP into ATP that we published previously (Fig. 1)^19^. The efficiency of the enzymatic reaction allows us to avoid side reactions (keeping the system simple) and control reaction rate, for example by changing the catalyst concentration. We can thus ensure that the reaction is fast enough to overcome passive coarsening and slow enough to avoid spinodal decomposition^19,20^. In addition, partitioning of the kinase offers an insight into the location of the reaction. We analyze growth at a single-droplet level, making it possible to investigate the dynamics of individual membrane-less protocells.

Here, we show that droplets grow actively driven by the enzymatic reaction, leading to a significant increase in size. In some conditions, nucleation is preferred over growth and droplet count increases. The droplets exhibit a common growth profile that can be rationalized in terms of protein diffusion, triggered by the reaction. By isolating the contributions of active and passive processes to droplet size evolution, we find that our complex coacervate droplets do not undergo Ostwald ripening and can remain stable for observation for more than an hour. Finally, under the same environmental conditions, droplets of different compositions grow at different rates, paving the way for the design of evolvable protocells.

## Results

ATP-based coacervates have previously been studied as dynamic membrane-less protocells compatible with growth, enzymatic reactions, and RNA partitioning^21,22^. Inspired by the phosphorylation-mediated liquid–liquid phase separation of peptides and RNA developed by the group of Keating^23^, our group achieved reversible ATP-poly-l-lysine coacervates with the introduction of pyruvate kinase (PyK) to generate ATP in situ from ADP and phosphoenolpyruvate (PEP)^19^. With the high efficiency of the PyK reaction and lack of side reactions that can overcomplicate nonenzymatic systems, we hypothesized we could achieve enough control of coacervation to obtain a coordinated behaviour like growth (see scheme in Fig. 1). In comparison to our previous work, we replaced poly-l-lysine with K_72_ as a cationic fluorescent protein, which has already been used to form droplets with RNA^24^. K_72_ contains 72 repeats of the pentapeptide VPGKG (an elastin-like sequence)^25,26^ and is labeled with green fluorescent protein (GFP). It can form condensates at low concentrations with ATP, which can be easily monitored by fluorescence microscopy.

### Coacervation made active

**Fig. 2:**
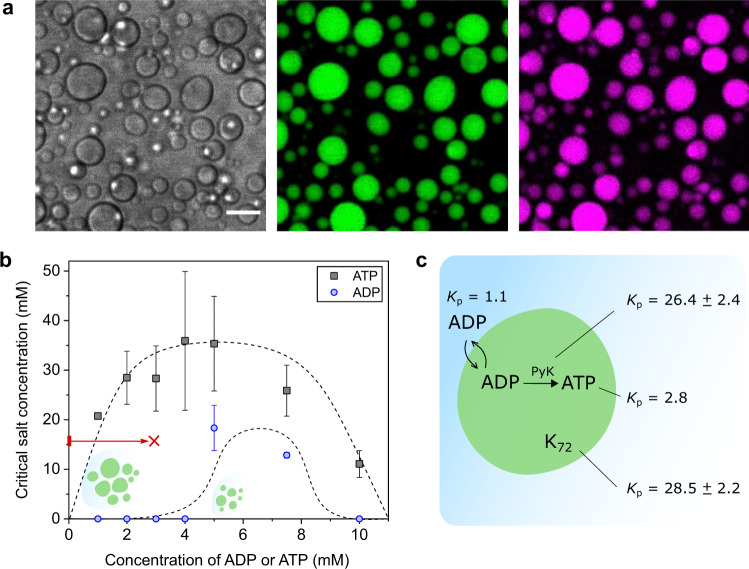
Main properties of ATP-K_72_ coacervate droplets. **a** ATP-K_72_ droplets containing Alexa Fluor-647-labeled pyruvate kinase. Channels are shown separately: gray (left)—transmission, green (middle)—GFP (attached to K_72_), magenta (right)—Alexa Fluor-647. K_72_ always contains the GFP tag; PyK was labeled with Alexa-647 only for this experiment. Scale bar: 10 μm. **b** The phase diagrams of ADP-K_72_ and ATP-K_72_ mixtures confirm that the conversion of ADP to ATP can induce coacervation under certain conditions and lead to growth (e.g., along the red line). The dashed lines representing the approximate phase boundaries are meant as a guide to the eye. Error bars represent one standard deviation of the intercept value in the linear fit used to determine critical salt concentration from a turbidity assay (see “Methods”, based on five turbidity measurements). Source data are provided in Supplementary Data 1. **c** Partitioning coefficients of the main components (measured via HPLC or fluorescence, see Supplementary Figs. 1 and 3).

The first step in the design of our system was to determine the conditions under which ATP (the droplet material), but not ADP (the substrate), forms droplets with the K_72_ protein (Fig. 2a). This coacervation window is the range of conditions where ATP-K_72_ droplets can nucleate and grow as a result of the conversion of ADP into ATP. By measuring the phase diagram in terms of salt concentration (Fig. 2b), we estimate the stability of coacervate droplets to a chemical reaction that produces charged by-products—in this case, the pyruvate kinase-catalyzed formation of ATP also generates pyruvate. The typical phase diagram of ADP/ATP-K_72_ complex coacervates shows that at 3 mM of nucleotide and no added salt, the difference between ADP and ATP in affinity for K_72_ is maximal, which is ideal to translate the progress of the chemical reaction into a volume change. We note that, when a reaction is introduced, the dispersion will contain a mixture of ADP and ATP, as well as fuel and other reaction products. The equilibrium composition of both phases in such a multicomponent mixture will deviate from the binodal lines of pure ADP-K_72_ or pure ATP-K_72_, most likely passing by intermediate binodal lines which we have not characterized, but assume are in between the ones in Fig. 2b. Nevertheless, our aim is to measure how droplets nucleate once a threshold ATP concentration is reached and grow via the formation of additional ATP, for which the selected starting concentrations in Fig. 2b are suitable.

We further determined the partitioning coefficient (*K*_p_) of the main reaction species to create a kinetic map of our system (Fig. 2c). We prepared host droplets of ATP-K_72_ at the same conditions as for the phase diagram, and added labeled pyruvate kinase and ADP (the latter at 3 mM, i.e., below the ADP-K_72_ binodal) as client molecules. As expected, the *K*_p_ of ATP (2.8) is higher than that of ADP (1.1), but even above the critical salt concentration of pure ADP-K_72_ mixtures, ADP can be incorporated as a client in ATP-K_72_ coacervates (see the phase compositions in Supplementary Fig. 1). To determine enzyme *K*_p_, we labeled it with Alexa Fluor-647 maleimide, targeting exposed cysteines. We chose a cysteine-reactive label to avoid modification of charged residues (lysines), which can affect the partitioning (Supplementary Figs.  2 and 3). Based on the measured partitioning coefficients (Fig. 2c), and the fast fluorescence recovery (Supplementary Fig. 4), we can make the following assumptions: (i) ADP can enter the droplets if they become depleted of it; (ii) ATP, PyK, and K_72_ accumulate inside the droplets and can exchange with the surroundings; and (iii) the reaction can occur inside the droplets, where the enzyme is concentrated. These are key requirements to keep the system out of equilibrium with a supply of substrate and attain reaction-driven growth.

### Single-droplet analysis of coacervates over time

After mapping out the conditions under which active droplets could exist, we investigated if a fuel-driven reaction could bring about active growth as a step towards evolvable protocells. Taking advantage of the fluorescence from the K_72_ proteins condensed inside the coacervates, we can monitor the evolution of individual coacervates nucleating, growing, and resting on a plane above the glass surface for at least an hour with confocal laser scanning microscopy (Supplementary Fig. 5 and Supplementary Data 2). To gain a fitness advantage, actively growing protocells must be able to overcome passive coarsening, occurring through coalescence or Ostwald ripening. We first compared passive, pre-formed ATP-K_72_ droplets to active droplets growing by conversion of ADP into ATP. In our setup, by directly tracking droplet size, fusion events are not mistaken for growth, but it remains important to establish the conditions under which active growth can outcompete passive coarsening. We detected the droplets by their boundaries and extract properties such as area, centroid position, circularity, and total fluorescence intensity. We labeled droplets by their centroid and then built a profile of radius over time, where each droplet has its own curve (Supplementary Fig. 6).

**Fig. 3:**
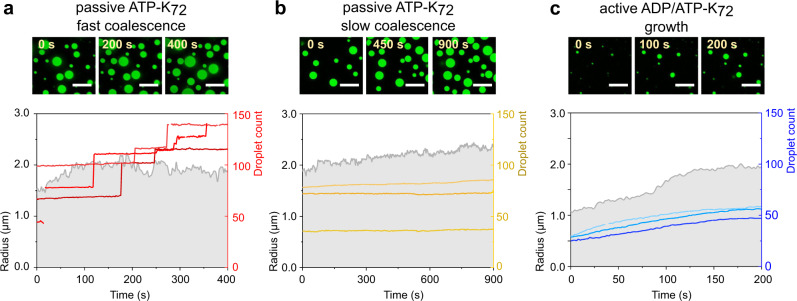
Passive and active droplets in radius profiles. Bottom: **a** Passive coacervate droplets exhibit discrete increases in radius or (**b**) at lower volume fraction can remain stable for minutes. **c** The gradual increase in droplet radius over time is characteristic of active droplets, for which also the droplet count increases. All: left axes indicate droplet radius (in μm) and right axes indicate droplet count. For visual clarity, only three exemplary traces were chosen out of each experiment. The output of the analysis containing all traces in (**a**) and (**b**) can be found in Supplementary Fig. 7. All traces of (**c**) are shown and discussed further in Fig. 5a. Top: scale bars are 10 μm. Full microscopy frames can be found in Supplementary Fig. 8. From the confocal slices, we extract droplet radii to calculate the total coacervate volume and use the field of view as a rectangular cuboid to calculate total volume.

A high-volume-fraction passive system can be achieved at a high poly-electrolyte concentration. At 3 mM ATP, 20 μM K_72_, we estimated the volume fraction based on centrifugation to be ca. 1%, a value in the same order of magnitude as when calculated from the microscopy images. At this volume fraction, most droplets exhibited steps in the radius profile (Fig. 3a). Frequent coalescence events can lead to discrete increases in droplet volume of tens of fL (μm^3^) every hour^17^, although the droplet count does not decrease due to simultaneous gravitational settling from the top of the solution to the glass plane.

At a lower concentration, and therefore lower droplet density (1 mM ATP, 20 μM K_72_), most passive droplets showed a stable size (Fig. 3b) that can persist for an hour (Supplementary Fig. 7). We observed significantly fewer coalescence events, as expected, but surprisingly, we also observed no measurable Ostwald ripening in the form of gradual expansion of large droplets and shrinkage of small droplets, even though we expected clear ripening according to our most conservative estimates of the ripening parameters (Supplementary Table 2). The absence of Ostwald ripening, which we explain in more detail in the following section, is a remarkable behaviour and of great importance for our goal to achieve active growth in very small coacervate droplets.

Based on our findings with passive droplets, we were hopeful to observe distinctly different kinetic traces for active droplets at low volume fractions. For ATP-K_72_ droplets forming by chemical conversion from ADP, the initial volume fraction is even smaller than that in Fig. 3b. Coalescence will therefore be even less frequent and is not expected to mask the onset of active growth. Indeed, the profiles of active growth (Fig. 3c) are clearly distinct from the two sets of passive profiles (Fig. 3a, b). When the ADP-K_72_ mixture was placed under the confocal microscope and fueled with PEP, droplets of 0.5-μm radius started forming within a minute. Especially at the initial times, the vast majority exhibited a continuous growth curve (Fig. 3c). Importantly, in contrast to passive droplets coarsening, growth does not compromise persistence and the droplet count, in this case, can increase (as shown in Fig. 3c and Supplementary Fig. 9).

### Suppressed Ostwald ripening of complex coacervate droplets

We noticed that—surprisingly—the active and passive droplets did not show any ripening, despite prolonged observation. Instead, droplets were found to remain stable for at least an hour. Ostwald ripening has been predicted to be suppressed in active emulsions, where a chemical reaction (with the appropriate rate) causes a bigger efflux of molecules from large droplets than from small ones^27^. Experimentally, however, active oily droplets coupled to an anhydride formation reaction exhibited accelerated ripening, as the reaction activation/deactivation flux adds to the diffusive flux between droplets^9^. In our experiments with either passive or active coacervate droplets, we did not observe any shrinkage of small droplets, suggesting that Ostwald ripening is being slowed down or prevented by an opposing force closely linked to the nature of our droplets^28^.

**Fig. 4:**
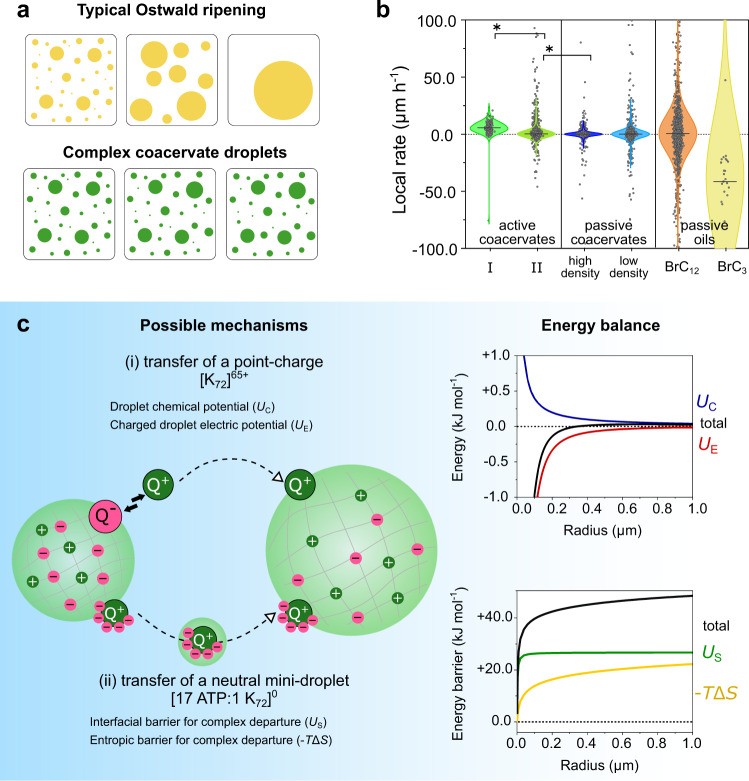
Ostwald ripening in complex coacervates. **a** Schematics of the distinct behaviour observed for oil-based droplets and complex coacervate droplets. **b** The local rates can be used to quantify that distinction: active coacervates grow (phases I and II are discussed below in Fig. 5), and passive coacervates (high or low droplet densities) remain stable in size; the shrinkage of some droplets of 1-bromododecane (BrC_12_) and 1-bromopropane (BrC_3_) can be detected in our method as negative local rate values. Although the median growth rate for BrC_12_ is positive, we see a significant fraction of droplets with a negative value, unlike the results for coacervates. Active and passive coacervates are significantly different (*) in a Mood’s median test (*P* < 0.05). Source data are provided in Supplementary Data 1. A complete overview of experimental conditions and sample size can be found in Supplementary Table 1. **c** Rationalization of suppressed Ostwald ripening in complex coacervates, taking into account two possible mechanisms: in (i), free poly-electrolytes, in this case, K_72_, are transferred, leaving an oppositely charged droplet. In (ii), coarsening happens through the transfer of electroneutral complexes from small to larger droplets. This complex has an interfacial area, represented by the green droplet encasing it. Next to each mechanism, the energy balance estimation for each of the mechanisms envisioned.

To understand why these complex coacervate droplets would not show ripening, we consider the balance of thermodynamic forces underlying Ostwald ripening. We estimate a lower limit for the expected rate of ripening based on the solubility of the least soluble droplet component, which in our case, is K_72_ with a saturation concentration of about 5 μM. Assuming a molecular volume of ca. 65 nm^3^ for K_72_^29^, we expect that the ATP-K_72_ complex coacervate droplets could show an increase in mean radius of 3–7 μm due to ripening per hour (see estimation in Supplementary Table 2)^17^. However, the droplets tracked have a radius of 0.4–3 μm, and analysis of their size over time, local growth rates, size–rate correlation, and droplet count do not agree with a ripening profile, suggesting that ripening is not only slow, but suppressed. We verified that this was not a limitation of our experimental setup by performing positive controls with passive oil droplets of 1-bromo-dodecane and 1-bromo-propane, for which we were able to visualize shrinkage (depicted as a negative growth rate in our analysis) and decaying droplet count (Fig. 4b).

Therefore, we hypothesize that complex coacervates are special liquids that exhibit suppressed Ostwald ripening, because they are formed via associative phase separation. Typical ripening is driven by the increased Laplace pressure inside small droplets (*U*_C_ in Fig. 4c), but ignores the energy associated with either the disruption of attractive interactions when charged molecules are removed from the droplet, or the entropy and interfacial energy involved in removing an electroneutral complex of one or more K_72_ molecules bound to ATP from the droplet. While it is hard to determine which of these mechanisms is at play when we observe no ripening, we show that in both cases, the associated energy or energy barrier can be large enough to prevent ripening.

For complex coacervates such as K_72_-ATP, which contain small molecules and proteins with relatively low charge densities, and which include additional salt, the droplet components are likely unpaired in the dilute phase^30^. We, therefore, consider the removal and transfer of the least soluble component, K_72_, as a separate species as a key step in coarsening. The separation of a positively charged K_72_ (*Q* = + 65*e*) from a coacervate droplet of size *R* will leave a residual negative surface charge density of $-Q/4\pi {R}^{2}$, which comes with an electrostatic penalty that is larger for smaller droplets (*U*_E_ in Fig. 4c). Weighing that penalty against the Laplace pressure difference that drives Ostwald ripening, we find that the exchange of material between complex coacervate droplets may not necessarily occur in the direction from small to large droplets. With typical estimates of the surface tension, molecular volume, and Debye length in our ATP-K_72_ coacervate droplets, the transfer of charged material from one droplet to another is endergonic regardless of the relative radii (Fig. 4c—mechanism i and Supplementary Table 2). Moreover, with many protein condensates carrying a small net surface charge^31^, the transfer becomes even more restricted, either because of electrostatic attraction at the source droplet, or repulsion at the target droplet.

In the alternative pathway, removal of an electroneutral complex from the coacervate in the form of a minidroplet of coacervate phase containing one or several positively charged K_72_ with roughly 17 ATP molecules per K_72_ is associated with an entropy loss of $-\Delta S/k={\rm{ln}}{\big({V}_{{\rm{drop}}}/{V}_{{\rm{complex}}}\big)}^{1/2}$, assuming that K_72_ and ATP were able to move freely throughout the liquid coacervate droplet before they were removed (*−T*Δ*S* in Fig. 4c). In addition, it can be argued that the complex has an interfacial energy of the order of $\gamma {V}_{{\rm{complex}}}^{2/3}$ (*U*_S_ in Fig. 4c). While both contributions, combined with the favourable uptake of the complex by a large droplet, do not result in an endergonic transfer, we do find that each of these contributions alone imposes a prohibitively large energy barrier on the Laplace pressure-driven ripening beyond a hypothetical droplet size of 5–10 nm (Fig. 4c—mechanism ii and Supplementary Table 2). This implies that, in this scenario, coarsening would be effectively suppressed by slowing it down to a negligible rate.

Our analysis suggests that Ostwald ripening can be effectively suppressed by the nature of the interactions underlying droplet formation and that many complex coacervates can persist for extensive times, provided that the charge of the building blocks is large enough. Indeed, our experiments indicate that Ostwald ripening is absent in both passive and active complex coacervate droplets, and we confirmed that Ostwald ripening was also absent in another, passive complex coacervate system (Supplementary Fig. 10). Importantly, from a protocell perspective, this means that if we introduce an active process in these slow- or non-ripening and slow-fusing droplets, the resulting active droplets could mimic cellular growth without interference from passive coarsening processes, and the growth can be controlled by the same parameters that control a chemical reaction.

### Growth at a single-droplet level

**Fig. 5:**
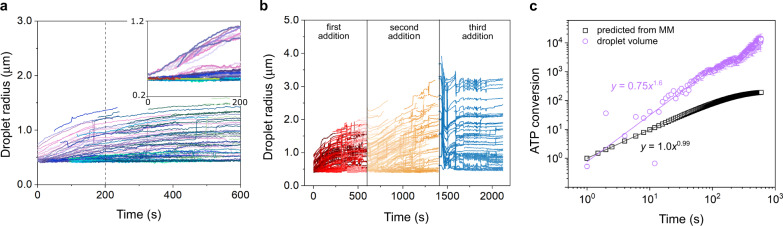
Growth of active droplets. **a** Radius traces of all droplets in a selected active droplet experiment (original: video 6). In the inset, the curves were shifted horizontally for better visualization of common behaviour. **b** Stepwise addition of fuel (PEP) to active droplets. In each step, 1 mM of PEP was added, after the growth curve plateau was reached. Original videos: 9, 10, and 11, respectively. Source videos are provided in Supplementary Data 1. A complete overview of experimental conditions and sample size can be found in Supplementary Table 1. **c** Profile of the ATP conversion based on average droplet volume evolution (calculated from the dataset in (**a**)), compared to the profile estimated based on Michaelis–Menten kinetics in solution, using *k*_2_ of 0.3 min^−1^ and ADP starting concentration of 3 mM. The solid lines are power-law fits to the initial 60 seconds of growth (*R*^2^ > 0.9, outliers not included). The calculated conversions have been normalized such that the initial slopes cross at (1,1). Note that the ATP conversion in growing droplets and solution cannot be compared directly, since the exact droplet volume fraction is not known.

Having established that ATP-K_72_ complex coacervate droplets show negligible Ostwald ripening on the timescale of our interest, we return to the active droplets of Fig. 3c to obtain a better understanding of the active growth. We found that the droplets start growing only after the addition of the pyruvate kinase’s second substrate or fuel, phosphoenolpyruvate (PEP), and that they grow significantly over the course of an experiment. A typical growth curve has two regions: initial fast growth, seemingly of a linear increase of radius with time; after around 5 min growth slows down, and after 10 min most droplets have reached a plateau of stable size, as can be seen in Fig. 5a (extended curves in Supplementary Fig. 11). The plateau coincides with the depletion of fuel, as predicted based on HPLC measurements of nucleotide concentration (Supplementary Fig. 12); indeed, if fuel is re-supplied, the droplets can regain growth (Fig. 5b). At the third consecutive addition of fuel, we did not observe significant growth, presumably because the system approaches, at least locally, the concentrated branch of the phase diagram, due to the accumulation of reaction products.

At a first glance, each droplet seems to have a unique trace, but that is mainly caused by the polydispersity in droplet size. All curves have the same overall shape and if horizontally shifted, two profiles become evident: nongrowing droplets and droplets growing with a common profile (inset in Fig. 5a), which is an indication that a common chemical mechanism underlies the growth. Droplets of small starting radii (*R* < 0.5 µm) that show a separate group of traces and are always delayed (i.e., they start to grow when their size exceeds the 0.5-µm threshold radius). This delay becomes more evident at lower enzyme concentrations (Supplementary Fig. 13), suggesting that these small droplets might lack any enzyme at all and rely solely on the incorporation of ATP produced in the dilute solution. Indeed, if we estimate the inner enzyme concentration based on a total enzyme concentration of 0.42 µM, a *K*_p_ of ca. 20, and a 1% volume fraction of droplets, the average number of enzymes in a 0.5-µm radius droplet is 2. Once these droplets surpass a threshold size at which they contain a higher enzyme count, they could start to grow more rapidly and their radius increases close to linearly in time.

To explain the observed growth profile, we consider the kinetics involved in droplet nucleation and growth. Once the first droplets are formed by nucleation (or if we add a small amount of pre-existing ATP-K_72_ droplets), the reaction can happen in two phases: droplets and surroundings. We assume that the rate of ATP production in the droplet is higher than in the surrounding solution, based on the measured ADP and PyK partitioning (Fig. 2c) and HPLC measurements of PyK kinetics in the presence of coacervates^32^, which show that the effective *k*_cat_ of PyK in a coacervate dispersion is the same as in a solution. The high inner ADP and PyK concentrations would be sufficient for a faster reaction in the droplets, and the conversion of ADP into ATP inside the droplets results in a continuous replenishment of ADP and uptake of additional K_72_ and PyK to maintain partitioning equilibrium. If transport of those compounds would be fast compared to the reaction, we expect the amount of new ATP produced to be directly proportional to the actual volume of the coacervate droplet, leading to an exponential increase in droplet volume (and radius) in time, analogous to the kinetics of a pure autocatalytic reaction^33^. However, in our case the droplet size does not increase exponentially in time, suggesting that transport of building blocks from the surroundings into the droplet is limiting the growth.

Of all building blocks, K_72_ and PyK are the largest compounds, present at relatively low concentrations compared to ADP, and the slowest to diffuse. As K_72_ is required as droplet material to compensate for the excess charges of ATP produced inside the droplets, we reason that transport of K_72_ limits the growth of droplets. The flux of molecules across the interface is proportional to the surface area ($4\pi {R}^{2}$) and the concentration gradient at the interface (d[K_72_]/d*R*). This situation is analogous to the growth of condensed cloud droplets in a saturated vapor phase, and the radial growth is predicted to follow: $R\left(t\right)={\left({R}_{0}+2\xi t\right)}^{1/2}$ after nucleation, where *ξ* is a function of the supersaturation of the environment, which is set in our case by the concentration of K_72_ in solution and the reaction rate^34^. For simplicity, we assume that *ξ* is constant in a short interval of time, and we find that the droplet volume will increase as $V\left(t\right)={\left(4\pi /3\right)\left({R}_{0}+2\xi t\right)}^{3/2}$, in agreement with our results in Fig. 5c, where we obtained an exponent of 1.61 ± 0.06. In short, the active droplets in our experiments grow as a result of an autocatalytic conversion of ADP into ATP, but the overall growth is limited by the diffusion of K_72_ from the surrounding solution to the droplet interface, where it can be taken up. We note that transport of other compounds, including PyK and PEP, could also limit the growth when their concentrations are altered. However, this would only change the growth rate constant *ξ* and not change the scaling of droplet size in time, as these compounds must also be transported by diffusion to the droplet interface^5,27^.

### Growth at a population level

In order to corroborate our model and analyze the effects of varying the concentrations of fuel, catalyst, and building blocks, we need to quantify the typical growth rate (the fitness) of an entire population of droplets. Since the droplets vary in size but show a universal growth profile (Fig. 5a), we chose to average their local growth rates, defined as the derivative of the radius versus time curve across a time interval, given in units of μm h^−1^. The derivative is calculated using a linear approximation over small intervals of 20 s, during the first 2 min of the reaction. We analyzed hundreds of droplets together in every experiment and found that also at the population level, active droplets have a distinct behaviour from passive droplets. The distance to neighbouring droplets, position in the well and droplet size (past a threshold) do not affect the droplet growth rate (Supplementary Figs. 14–17).

**Fig. 6:**
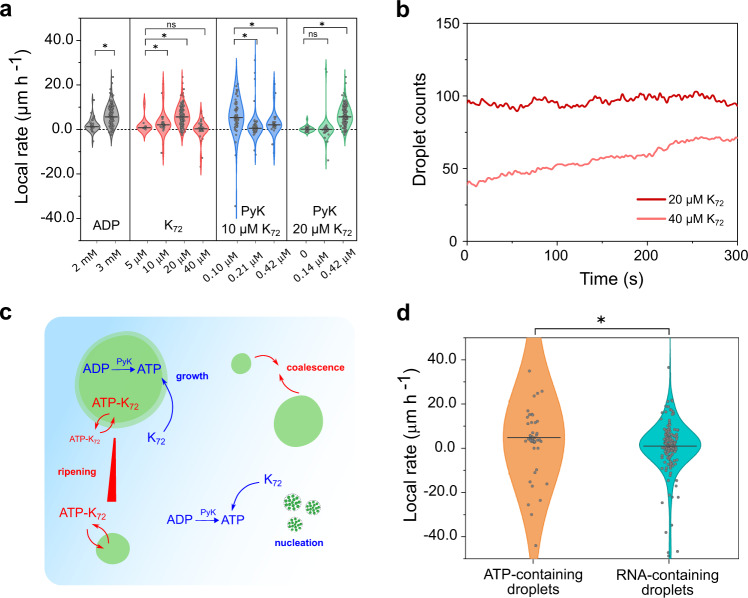
Growth rate of active droplets. **a** Growth rate dependence on different reaction–diffusion conditions. The local rate was measured for all droplets in a frame within 200 s of the experiment. In different experiments, the concentration of ADP, K_72_, and PyK was varied. Median growth rate differences were evaluated as significant (*) or nonsignificant (ns) in a Mood’s median test (*P* < 0.05). Source data are provided in Supplementary Data 1. A complete overview of conditions and sample size can be found in Supplementary Table 1. **b** Droplet count during the growth phase of two of the experiments depicted in (**a**). **c** Active droplets undergo the processes in blue: they grow around seeding droplets or also nucleate in a supersaturated solution of K_72_. The passive processes in red—ripening and coalescence—are suppressed or minimized in our system. **d** Active droplets of different compositions grow at significantly different rates (*) in a Mood’s median test (*P* < 0.05). Source data are provided in Supplementary Data 1.

We varied reaction and diffusion conditions as shown in Fig. 6a. Active droplets formed from 2 mM substrate (ADP) grew 20× faster than passive droplets (1.24 versus 0.06 μm h^−1^, see Supplementary Table 1); droplets could grow 100× faster than passive droplets when ADP is increased to 3 mM. Higher K_72_ concentrations indeed accelerated growth, but at 40 μM there was a reversal in the effect, which we attribute to a rising droplet count (Fig. 6b). The increase in droplet count, although also a feature of an active system, competes with growth. Similarly, when protein concentration was low (10 μM K_72_), we observed a maximal growth rate at the lowest enzyme concentration tested. The increase in enzyme concentration from 0.10 to 0.42 μM was also accompanied by an increase in the initial number of droplets, that we cannot control in our setup. The solution reaches supersaturation more rapidly, which facilitates widespread nucleation of multiple nuclei that then grow limited by diffusion, rather than growth of some seeding droplets, and the measured growth rate is lower^34^. When the enzyme concentration was varied and the protein concentration was higher (20 μM K_72_), the optimal enzyme concentration for growth also shifted to a higher value (0.42 μM). The complex balance between the two phases, and the two processes (reaction and diffusion), may result in two distinct active droplet regimes—nucleation-dominated or growth-dominated—but both are relevant as protocell models (Fig. 6c).

The fact that we obtain significantly different growth rates by varying substrate, catalyst, or building block concentration means that our protocell model can have different fitness depending on its composition and the environmental conditions. This is crucial for research aiming to achieve Darwinian evolution with populations of artificial cells^35,36^. We tested this feature by subjecting two different populations to the same environmental conditions: one composed of K_72_, ADP, and a seeding concentration of ATP, enough to have droplets from the start; and another mixture where the seeding ATP was replaced by an RNA oligomer ((ACGU)_6_), which also phase separates with K_72_ (Supplementary Fig. 18a). As in the case of ATP-K_72_ droplets, the enzyme PyK has a high partitioning in the RNA-containing droplets (*K*_p_ = 49.4, Supplementary Fig. 18a), but RNA, with a *K*_p_ of 18, displaces ADP in the droplets^37^, so we expected lower growth rates. Indeed, although the RNA droplets started larger, they grew at 5x lower rates than the ATP-only droplets (Fig. 6d). RNA-containing droplets could be designed to grow faster by using an enzyme with a higher preference for RNA droplets, or by making use of RNA’s catalytic capacity^8,38^.

## Discussion

We developed a protocell model that mimics two key features of cellular growth: the volume expansion with a constant protocell count and the intrinsic relation between content and size. The ATP-K_72_ coacervates grow as a result of a reaction that converts ADP into droplet-forming ATP, catalyzed by pyruvate kinase. The catalyst is an important component, that due to its efficiency and lack of side reactions, allows for fine control of ATP formation. Although the use of an enzyme may seem to decrease the prebiotic relevance of our model, we argue that the active coacervate droplets do not rely on any specific interaction and the principles found here can be applied to any complex coacervate.

Several works have pointed out the lack of a membrane as a disadvantage of coacervates as protocellular models^39,40^. Indeed membrane-less droplets are prone to (accelerated) Ostwald ripening and have no barrier to prevent fusion, but we found that complex coacervate droplets are remarkably stable. Unlike commonly studied emulsions, complex coacervate droplets are held together by attractive electrostatic interactions. We show that the magnitude of the electric attraction between a droplet and a departing soluble component like K_72_ may compensate for the driving force of Laplace pressure from small to larger droplets. Alternatively, the departure of an electroneutral, soluble complex of ATP-K_72_ faces a high-energy barrier due to the creation of an additional interface and the decrease in entropy required. Therefore, complex coacervate emulsions can remain stable for hours without showing any sign of Ostwald ripening. More than a technical advantage that allows us to measure growth rates without the competition of ripening, this is a requirement for a growing protocell—before replicators and reproducers, there must be survivors^41^.

An advantage of our approach is that we are able to follow individual droplets. This allows to separate the contribution of (rare) fusion events from steady, active growth; and additionally, to obtain a precise profile of droplet sizes and to evaluate the influence of reaction rates and environmental factors on the growth rate of droplets. Most active droplet studies so far have focused on droplet count and average size, which are more susceptible to the interference of droplet motion^15,42^. Based on individual droplet traces, we found that our fuel-driven active droplet grows by diffusion, in a classical nucleation-growth fashion^34^, but that the rate is determined by the ATP-forming reaction. As a result, droplet radius has a *t*^1/2^ dependency, and the speed can be controlled by substrate, catalyst, and protein concentrations. Moreover, the growth profile shows that liquid–liquid phase separation alters the overall kinetics of the kinase reaction, by introducing positive feedback where larger droplets have an increased enzyme and ADP copy number, similar to the effect of physical autocatalysis^43^.

Growth and survival are, ultimately, properties of a population, and we show that we can use our model system to create populations with distinct growth rates, which can lead to distinct fitness. From microscopy experiments where the droplets do not need to be immobilized or stabilized, we extract growth rates of all droplets in both populations and found that RNA-containing droplets grow 5x more slowly than the original ATP-K_72_ droplets, which can be rationalized in terms of the partitioning of ADP and therefore, the strength of the positive feedback in the kinase reaction. We point out that the eventual slowing down of growth is not an intrinsic property of active coacervates, but a consequence of the limited amount of K_72_ and PyK. We envision that by designing systems with higher catalytic efficiency in the presence of RNA, and by introducing a common substrate supply, this is the first step toward competition and evolution of active coacervate protocells.

## Methods

### Materials and solution compositions

For the coacervates preparation, magnesium chloride anhydrous, sodium chloride, ATP disodium salt, ADP disodium salt, and pyruvate kinase type VII from rabbit muscle (EC 2.7.1.40, 2.8 mg mL^−1^, ca. 1400 units mL^−1^, molecular weight used: 223 kDa—tetramer) were purchased from Sigma-Aldrich; Cy5-(ACGU)_6_ RNA oligomer was purchased from Integrated DNA Technologies; HEPES free acid and phosphoenolpyruvate monopotassium salt were purchased from FluoroChem. For the microscopy chambers: methoxy-PEG silane (MW 5000) was purchased from JenKem Technology USA and 8- or 18-wells chambered μ-slides with glass bottom (No. 1.5 polymer coverslip) were acquired from Ibidi. For enzyme labeling, Alexa Fluor-647 C_2_ maleimide was purchased from Fischer Scientific. For HPLC experiments, potassium phosphate mono and dibasic salts were purchased from Sigma-Aldrich.

The following stock solutions were prepared by dissolving or diluting in MilliQ: 500 mM and 100 mM HEPES pH 7.4 (adjusted with NaOH 6 M), 10 mM MgCl_2_, 1 M NaCl, 100 mM ADP, 100 mM ATP, pyruvate kinase 1 mg mL^−1^. A 100 mM PEP solution was prepared in the 500 mM HEPES. All of the latter were stored at −20 °C for no longer than a month. mPEG silane was dissolved and sonicated in dry DMSO to a 30 mg mL^−1^ concentration, and the stock kept for no longer than a week at room temperature. Alexa Fluor-647 NHS ester was dissolved in dry DMF to a concentration of 10 mg mL^−1^ and kept at −20 °C.

### Pyruvate kinase labeling

We followed Thermo-Fischer instructions: 100 μL of enzyme stock, directly as purchased (PyK 2.8 mg mL^−1^ or ca. 12 μM), were mixed with 100 μL of HEPES 0.1 M to reach pH 7 and a concentration of ca. 6 μM. Disulfide bonds were reduced by adding a large excess of DTT (2 μL of a 0.1 M stock); the excess was removed after 30 min by centrifugal filtering (MWCO 3 kDa, 2 mL, Centricon, Merck) with degassed HEPES buffer, until the volume reached ca. 200 μL again. Alexa Fluor-647 C_2_ maleimide was freshly dissolved in DMF (10 mg mL^−1^ or 7.7 mM stock) and 1.5 μL were added to the mixture (final 60 μM of dye, or 10 equiv. in regards to PyK tetramer). The mixture was placed on a thermoshaker for 2 h, at 600 rpm and room temperature (ca. 21 °C). For removal of unreacted dye, the reaction mixture was diluted to 2 mL with phosphate buffer (20 mM, pH 7) and transferred to a previously wetted centrifugal filter (MWCO 3 kDa, 2 mL, Centricon, Merck). Following fabricator instructions, the mixture was centrifuged at 500 × *g* for 30 min at 4 °C. Until the filtrate was colorless and 50 μL in volume, the following steps were repeated: re-suspend with a pipette, dilute to 2 mL with phosphate buffer, and centrifuge. The flow-through was kept for control experiments, and the enzyme solution was further purified by dialysis against 14 mL of MilliQ overnight (Thermo Scientific™ Slide-A-Lyzer™ MINI Dialysis Device, 3.5 K MWCO, 2 mL).

### Phase diagram

Coacervation of K_72_ and nucleotides ADP or ATP was always assessed with a commonly used turbidity assay, combined with microscopy. The absorbance at 600 nm was measured using a plate reader Spark M10 (Tecan), for samples containing: 25 mM HEPES pH 7.4, 20 μM K_72_, 1 mM MgCl_2_, and a varying concentration of ADP or ATP ranging from 1 to 10 mM. The samples were prepared on a 30-μL scale and placed in a 384-well plate (Nunc, flat bottom). Absorbance (Abs) was measured before and after 2 μL additions of NaCl 0.5 M, until it reached the value of the control lacking any nucleotide. Turbidity (%) was calculated as 100 (1 − 10^-Abs^). Critical salt concentration was calculated using the last three values of absorbance measured to extrapolate the concentration needed for Abs = 0 (relative to the control).

### Partitioning coefficients

Partitioning of K_72_, which always contains the GFP label, and of pyruvate kinase was calculated via confocal microscopy. The active coacervates were prepared in the default composition, and 1% volume of Alexa-647-labeled pyruvate kinase (as obtained after purification) was added to the mixture. The averaged intensity of GFP and Alexa-647 emission was calculated for multiple droplets. A blank for both channels was obtained with a sample containing only buffer, and the averaged intensity taken as background intensity. The partitioning coefficient of the protein or the enzyme was then calculated as *K*_p_ = (I_coacervate_ – I_background_)/(I_dilute phase_ – I_background_). *K*_p_ of labeled pyruvate kinase was considered to represent the *K*_p_ of un-labeled enzyme.

Partitioning of ADP, ATP, and PEP was measured using centrifugation and anion-exchange HPLC. Passive coacervates in their default composition were prepared, but now PEP and ADP were added as well (3 mM each), in a total volume of 100 μL. The sample was centrifuged for 30 min, after which the coacervate phase (cp) can be seen as a pellet at the bottom of the Eppendorf. The dilute phase (dp) was removed, avoiding as much as possible to collect the coacervate phase (cp) as well. The pellet was dissolved with 30 μL of NaCl 1 M, and then pipetted back to measure its volume. Both phases were then analyzed using a Shim-pack WAX-1 column (particle size 5 μM, 4.6 × 50 mm, Shimadzu), at 1 mL min^−1^ flow and 45 °C, using a gradient 0–100% B in 15 min (A: potassium phosphate buffer pH 7, 20 mM; B: potassium phosphate buffer pH 7, 480 mM). The peaks in the 254 nm chromatogram with retention times of 10.0 and 12.4 min were identified as ADP and ATP, respectively. The peak in the 215-nm chromatogram with a retention time 9.5 min corresponds to PEP. The partitioning coefficient was then calculated as *K*_p_ = peak_area_cp_ ×  dilution_factor_cp_/peak_area_dp_ × dilution_factor_dp_.

### Microscopy chambers preparation

The Ibidi μ-slides were functionalized with methoxy PEG to minimize splashing of the coacervate droplets and allow a more accurate measurement of radius over time. The protocol was adapted from Gidi et al.^44^ Methoxy-PEG silane (MW 5000) was added to dry DMSO (30 mg mL^−1^, ca. 20 μL per well to be functionalized) and placed in a thermoshaker at 60 °C. While it dissolved completely, the μ-slides were cleaned thoroughly: washed with dilute detergent, distilled water, and ethanol, and dried with pressurized air; then placed in a plasma cleaner (in a usual cleaning cycle according to fabricator instructions) or an ozone cleaner. This removes adsorbed particles, making all hydroxyl groups available for bonding with the PEG silane. The slide was then placed in the oven at 60 °C to prevent precipitation when the PEG silane solution comes into contact with the glass. Finally, the solution was added to each well, the slide was placed in a covered glass Petri dish, and the Petri dish inside an oven at 60 °C. After 2 h, the slide was washed thoroughly with ethanol, MilliQ water (with sonication for 5 min), and ethanol, then dried with pressurized air, and placed in an oven to dry completely. The slides were used the day after, for a maximum of 2 weeks, or surface defects start to be observed.

### Image and video acquisition

Images and time lapses were recorded at room temperature on a CSU X-1 Yokogawa spinning disc confocal unit connected to an Olympus IX81 inverted microscope, using a ×100 piezo-driven oil immersion objective (NA 1.3) and a 488 nm laser beam. Emission was measured at 500–550 nm, with 100 ms of exposure time, at a rate of 30 frames per minute, using an Andor iXon3 EM-CCD camera. The acquired images have a pixel size of 141 nm and a field of view of 72 × 72 μm^2^.

Indicated samples were recorded on a Liachroic SP8 confocal inverted microscope (Leica Microsystems, Germany) equipped with a DMi8 CS motorized stage, using the LAS X v.3.5 acquisition software and a ×20 air (0.75NA) or a ×10 air (0.45NA) objective, depending on the nature of the droplets. For the GFP channel, 0.6% of the nominal power of a cyan laser @488 nm and a normal PMT detector were used, measuring at 493–620 nm, with a gain of 600 V and an offset of −0.1%. For the Alexa-647 channel, 1.5% of the total power of a red laser @638 nm and HyD SP GaAsP detector in Standard mode acquiring at 658–779 nm were used. Images were acquired at a rate of 12–30 frames per minute and have a pixel size of 377 nm or 1.88 μm, depending on the objective.

### Active coacervates experiments

All samples were prepared just before an experiment, usually in a 20 μL size; the components were kept on ice during preparation, but not the mixture. Active coacervates had the default composition of (in order of addition): 50 mM HEPES pH 7.4, 0.5 mM MgCl_2_, 3 mM ADP, 20 μM K_72_, 0.42 μM pyruvate kinase, and 3 mM PEP. For investigating the effect of kinase activity, substrate concentration, and protein diffusion on the growth rate, the default concentrations were used, but the following were changed, respectively: the enzyme concentration was varied ranging from 0.1 to 0.42 μM, PEP was varied from 1 to 3 mM, or K_72_ was varied from 5 to 40 μM. A negative control without enzyme was performed. See Supplementary Table 1 for the full list of conditions.

### Passive coacervates and Ostwald ripening controls

Passive ATP-K_72_ coacervates were used as negative controls for growth, and contained (in order of addition): 50 mM HEPES pH 7.4, 1–3 mM ATP, 20 μM K_72_ and 0.5 mM MgCl_2_ The mixtures were prepared directly in the passivated microscopy chamber, and covered with a glass slide before recording 1-h long videos. Oil droplets were used as positive controls for Ostwald ripening, and prepared at 2% v/v fractions, in the presence of 2% v/v SDS and Nile Red as a fluorescent dye. We chose 1-bromo-dodecane and 1-bromo-propane based on their densities and solubilities.

### Competition assay

The two droplet populations were analyzed separately but prepared with the same enzyme and protein stocks. The reference population was based on our default system: 50 mM HEPES pH 7.4, 3 mM ADP, 20 μM K_72_ and 0.5 mM MgCl_2_, with the important difference of 1 mM ATP being added to pre-nucleate droplets. The second population was composed of: 50 mM HEPES pH 7.4, 3 mM ADP, 10 μM Cy_5_-(ACGU)_6_ RNA oligomer, 20 μM K_72_, and 0.5 mM MgCl_2_. Under these conditions, there are droplets before any ADP conversion.

### Pyruvate kinase activity

Enzyme activity in the presence of coacervates was determined by measuring ATP concentration in the emulsion as a whole, at different reaction times. Ten copies of the active coacervates (default composition) were prepared, and for each copy the reaction was quenched at a different time, using acetic acid (to pH 2, or 1% v/v). Conveniently, the low pH also dissolves the coacervates. The analysis was done by HPLC, using the same column and run as described in Partitioning coefficients. The control experiment was a sample of equal composition, with the addition of 100 mM NaCl to dissolve existing ADP-K_72_ coacervates, and prevent formation of ATP-K_72_ coacervates.

### Quantitative video analysis

Raw fluorescence confocal microscopy videos were processed and analyzed with MatLab 2019 Image Processing Toolbox. In brief, the script uses customized blurring and smoothing kernels to correct for background emission and prepare the video for edge detection; performs edge detection of objects on each frame with a canny operator, with thresholds customized per video; labels the objects based on their centroid and extracts area, circularity and pixel intensity. Across frames, the script compares centroids to distinguish between fusion, settling, and growing events. We select relevant droplets based on an aspect ratio <2.5 and on a minimum number of 30 frames accurately tracked.

The properties are then analyzed in a second pipeline that lists properties such as area, radius, volume, and pixel intensity, per droplet, and per frame. It also determines the slope of the radius versus time curve in intervals of ten frames (or 20 s in most cases), after outliers are removed with a moving average interpolation. Within that interval, a linear approximation is valid and the linear slope is taken as the local rate. This means that each droplet may have up to ten different local rates determined, but this parameter and method are a convenient and sufficient way to group droplets in the same experiment.

### K_72_ expression and purification

We adapted the procedure previously described by Pesce et al. and Te Brinke et al. as follows^24,25^. BL21(DE3) cells were transformed with the pET25-sFil-K_72_ plasmid. Expression was performed in Terrific Broth medium (TB; 12 g L^−1^ tryptone and 24 g L^−1^ yeast autolysate) enriched with phosphate buffer (2.31 g L^−1^ potassium phosphate monobasic and 12.54 g L^−1^ potassium phosphate dibasic), glycerol (4 mL per 1 L TB), glucose (0.1 wt%), and 100 µg mL^−1^ ampicillin. The TB was supplemented with 0.10 g of amino acids lysine and proline per 1 L of TB. The bacterial cultures were grown at 37 °C till an optical density OD_600_ reached saturation (1.5–1.8), subsequently, cells were cooled to 18 °C to allow expression overnight. Cells were pelleted at 5000 × *g* and resuspended in lysis buffer consisting of 10 mM Tris, 300 mM NaCl, 20 mM imidazole, pH 8, supplemented with 1× complete protease inhibitor cocktail (Roche). Cells were disrupted through sonication on ice and cleared by centrifugation at 20,000 × *g* at 4 °C.

His-tag labeled K_72_ was purified from the soluble fraction with a HisTrap column (GE Healthcare, elution buffer: 10 mM Tris pH 8, 300 mM NaCl, 500 mM imidazole). After dialysis against size exclusion (SEC) buffer (10 mM Tris pH 8, 300 mM NaCl), the protein was concentrated to 2–4 mL using a Vivaspin 15 concentrator (MWCO of 30 kDa). Then the protein was passed through a S200 SEC column (GE Healthcare). Protein purity was analyzed by SDS-PAGE using a 4–20% mini-Protean gel (Bio-Rad) stained with instant blue, pure K_72_ fractions with the corresponding size were combined and dialyzed against MilliQ. K_72_ stock solution was obtained by concentrating the protein using a Vivaspin 15 concentrator (MWCO 30 kDa) till the protein reached a concentration of 80 µM. Aliquots of the stock solution were snap-frozen and stored at −80 °C.

### Statistics and reproducibility

The local rate plots contain all slopes that could be determined from the linear approximation, at the time interval indicated in Supplementary Table 1; the actual number of droplets analyzed in each experiment can be found in the same table. Violin plots were built in OriginLab 2020, using a kernel-smooth distribution and scaled by width; data points are jittered for visualization and the median line is included (actual values in Supplementary Table 1). The difference between the results is significantly different (*) if *P* < 0.05 in a Mood’s median test. Representative microscopy images used for the determination of partitioning coefficients (Fig. 2 and Supplementary Figs. 3, 4, and 18) could be reproduced at least 3× with independent samples. Representative microscopy experiments used in the measurement of growth rates were performed in a serial variation of conditions, as shown in Supplementary Table 1, and all showed consistent active behaviour (Fig. 3 and Supplementary Fig. 8). In all cases, typically tens of droplets were tracked in each experiment, as indicated in Supplementary Table 1, and data of all droplets are shown in the corresponding growth rate distributions (Figs. 4 and 6).

### Reporting summary

Further information on research design is available in the Nature Research Reporting Summary linked to this article.

## Supplementary information

Supplementary information

Description of Additional Supplementary Files

Dataset 1

Dataset 2

Reporting Summary

## Data Availability

Source data are provided with this paper.

## Code availability

Codes for data analysis are provided as Supplementary files with this paper.
