# Supplementary material for: Active coacervate droplets are protocells that grow and resist Ostwald ripening
Source: Nat Commun. 2021 Jun 21;12:3819. doi: 10.1038/s41467-021-24111-x (PMC8217494; doi:10.1038/s41467-021-24111-x)
Supplement: Supplementary file 1 — Supplementary information [file 41467_2021_24111_MOESM1_ESM.pdf]

Supplementary information

Active coacervate droplets are protocells that grow and resist Ostwald ripening

Karina K. Nakashima[1], Merlijn H. I. van Haren[1], Alain A. M. André[1], Irina Robu[1] and Evan Spruijt[1*]

[1] Institute for Molecules and Materials, Radboud University, Heyendaalseweg 135, 6525 AJ Nijmegen, the Netherlands.

[*] Correspondence: e.spruijt@science.ru.nl

Table of Contents

I. Supplementary information to the section: Coacervation made active 2

II. Supplementary information to the section: Single-droplet analysis of coacervates over time . 4

III. Supplementary information to the section: Suppressed Ostwald ripening of complex coacervate droplets.. 7

IV. Supplementary information to the section: Growth at a single-droplet level 10

V. Supplementary information to the section: Growth at a population level 12

VI. Supplementary references 15

I. Supplementary information to the section: Coacervation made active

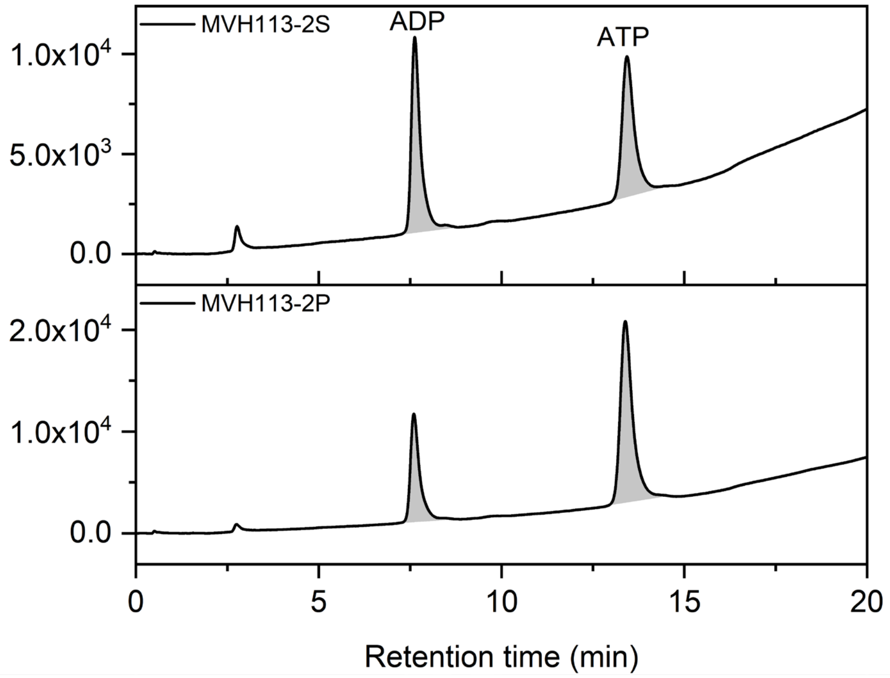

Supplementary figure 1. Analysis of ATP-K_{72} coacervates to which ADP was added as a client molecule. The sample is composed of 10 µM K_{72}, 3 mM ADP, 3 mM ATP, 50 mM HEPES pH 7.4 and 0.5 mM $MgCl_2$. At these buffer and salt conditions, ADP alone does not form droplets with K_{72} as it is above the critical salt concentration found in the phase diagram of main Figure 2B. In the presence of ATP however, we can detect ADP in both phases (dilute: MVH113-2S, coacervate: MVH113-2P). The chromatograms were measured at 254 nm after centrifuging the emulsion for 30 minutes. Each phase was then diluted 50X from the original. Peak areas were used in calculating K_p of ADP (1.1) and ATP (2.8) in ATP-K_{72} droplets.

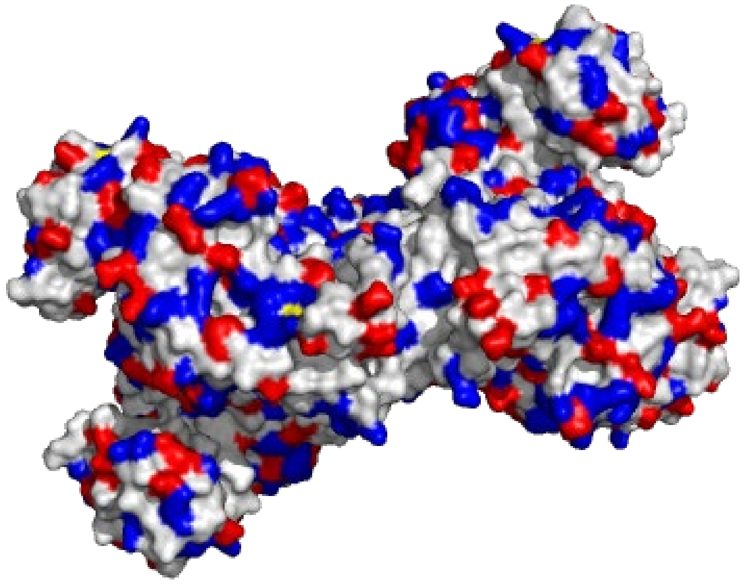

Supplementary figure 2. Exposed lysine (blue), glutamate (red) and cysteine (yellow) residues in a tetramer of recombinant rabbit muscle pyruvate kinase (PDB-1f3w). The abundance of charged patches suggests the protein can partition inside charge-based coacervates, and that lysine residues are relevant to its partitioning behaviour.

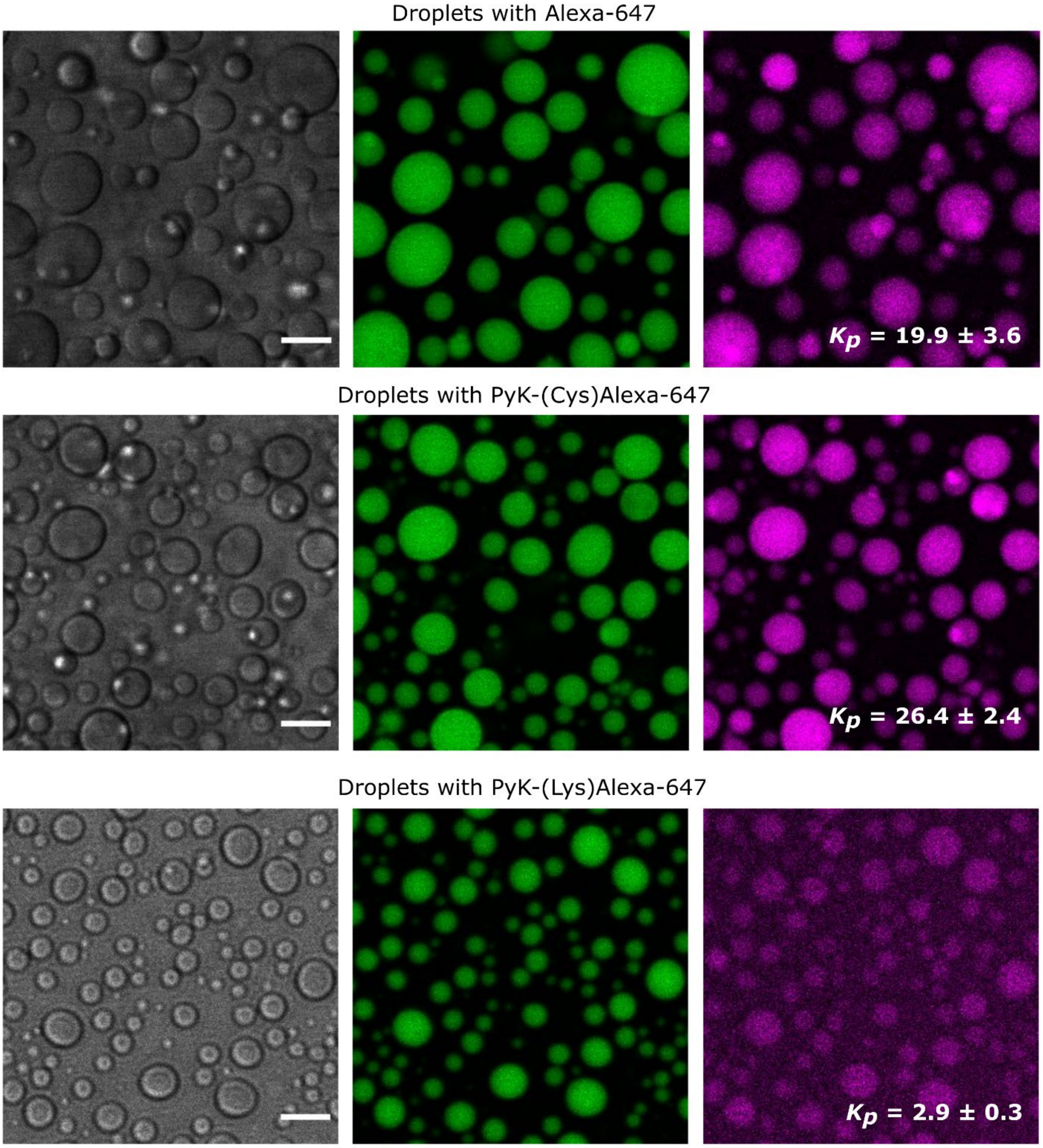

Supplementary figure 3. Micrographs used to determine partitioning coefficients (K_p). All mixtures contain 20 µM K_{72}, 3 mM ATP, 50 mM HEPES pH 7,4 and 0.5 mM $MgCl_2$, in addition to the labelled component indicated in the figure. Transmission is shown in gray LUT, emission at 488 nm excitation is shown in green and at 640 nm excitation, in magenta. Alexa-647 is the free dye in the flow-through obtained after the labelling reaction of pyruvate kinase and purification (see Methods). The K_p of K_{72} was determined to be 28.5 ± 2.2, from three samples and using five droplets near the center of the frame, discounting the blank intensity at 488 nm excitation. The K_p's of the dye and labelled enzymes were determined from the magenta channel, using five droplets near the center and discounting the blank intensity at 640 nm excitation. We took the K_p of PyK-(Cys)Alexa-647 as the most representative of the unmodified enzyme, as it is higher than that of the free dye. PyK-(Lys)Alexa-647 partitions much less as more than one lysine residue is labelled, decreasing the net surface charge of the protein. Images are shown before brightness adjustment. Scale bar: 10 µm.

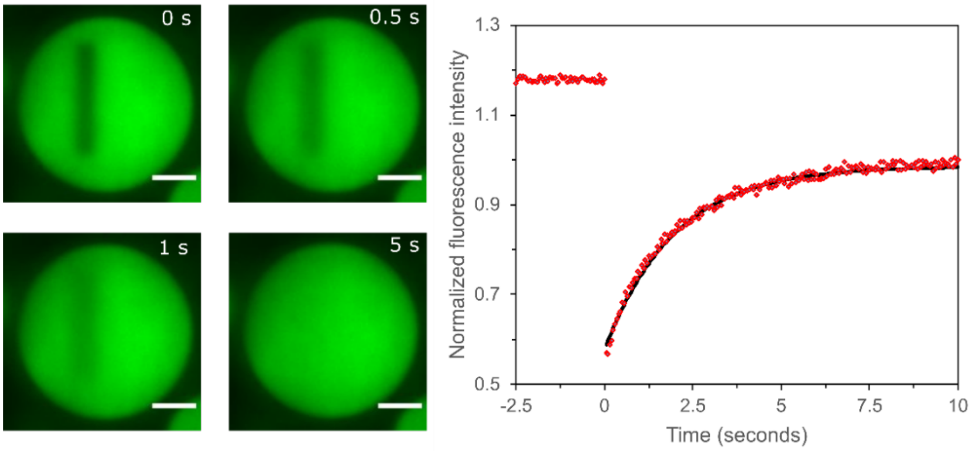

Supplementary figure 4. Fluorescence recovery after photobleaching (FRAP) of ATP-K_{72} droplets (typical passive coacervate composition, 3 mM ATP and 20 µM K_{72}). Scale bar: 5 µm.

II. Supplementary information to the section: Single-droplet analysis of coacervates over time

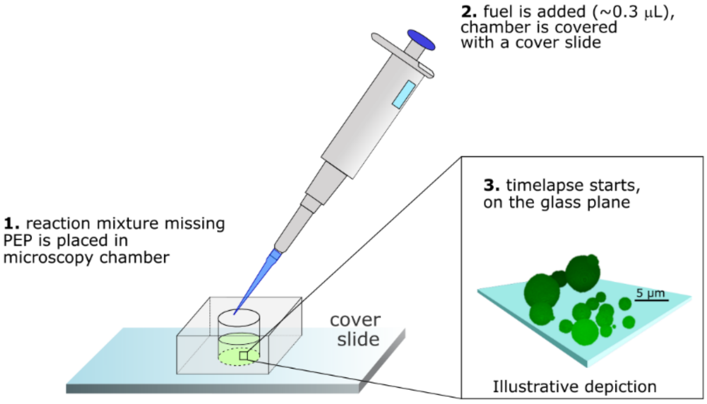

Supplementary figure 5. Scheme of the microscopy chambers used in active droplets experiments. The bottom cover slide (#1.5) is passivated with the PEGylation protocol described in Methods. The illustrative depiction is based on the z-stack provided as supplementary file.

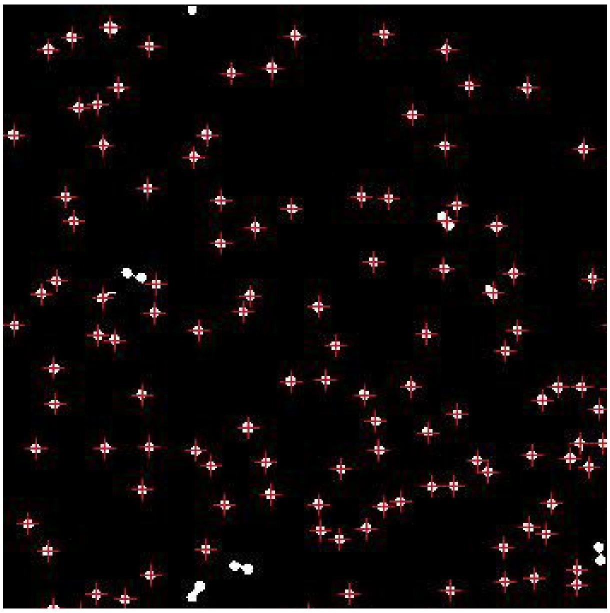

Supplementary figure 6. Example of edge-detected droplets, filled and labelled by their centroids. The video analysis used two MatLab based scripts available as Supplementary files.

Supplementary table 1. Short-name/experimental conditions correspondence for all videos analysed. Final concentrations in the microscopy chambers. Local rate median was determined from the distribution of local rates, or d*R*/d*t*, during the initial 200 s of recording, except when indicated otherwise.

Description	Short name	$[K_{72}]$ (µM)	$[ADP]_0$ (mM)	$[PEP]_0$ (mM)	[PyK] (µM)	$[ATP]_0$ (mM)	Average droplets detected	Local rate median (µm h^{-1})
PyK series 20 µM K_{72} no-reaction control	Video 3	20.0	3.0	3.0	0	-	6	0.06
K_{72} series	Video 4	5.0	3.0	3.0	0.42	-	42	0.74
	Video 5	10.0	3.0	3.0	0.42	-	28	2.09
	Video 6-I	20.0	3.0	3.0	0.42	-	58	5.63
	Video 6-II*[i]	20.0	3.0	3.0	0.42	-	80	0.27
	Video 7	40.0	3.0	3.0	0.42	-	3	0.23
PyK series 10 µM K_{72}	Video 8	10	3.0	3.0	0.21	-	44	0.50
Stepwise addition	Video 9	20.0	3.0	1.0	0.42	-	78	3.63
	Video 10	20.0	2.0	1.0	0.42	-	84	4.98
	Video 11	20.0	1.0	1.0	0.42	-	40	0.52
PyK series 10 µM K_{72}	Video 16	10.0	3.0	3.0	0.10	-	15	5.36
PyK series 20 µM K_{72}	Video 18	20.0	3.0	3.0	0.14	-	21	0.04
ADP series	Video 19	20.0	2.0	3.0	0.42	-	53	1.24
Passive coacervates – high density	Video 23[i]	20.0	-	-	-	3.0	118	0.0096
Passive coacervates – low density	Video 24[ii]	20.0	-	-	-	1.0	113	0.0004
Competition assay	Video 27	20.0	3.0	3.0	0.42	1.0	100	4.96
	Video 28	20.0	3.0	3.0	0.42	1.0 (+ 10 µM RNA)	19	0.99
Passive oil	Video 25[ii]	1-bromo-dodecane (2% v/v) and SDS (2% v/v)					71	0.63
Passive oil	Video 26[ii]	1-bromo-propane (2% v/v) and SDS (2% v/v)					8	-42.17
Complex coacervates	Video 29	0.30 M PDDA and PSPMA					-	-

Notes:
[i] *Refers to the interval of 300-900s of the video.*
[ii] *Refers to the entire video range of 30 minutes.*

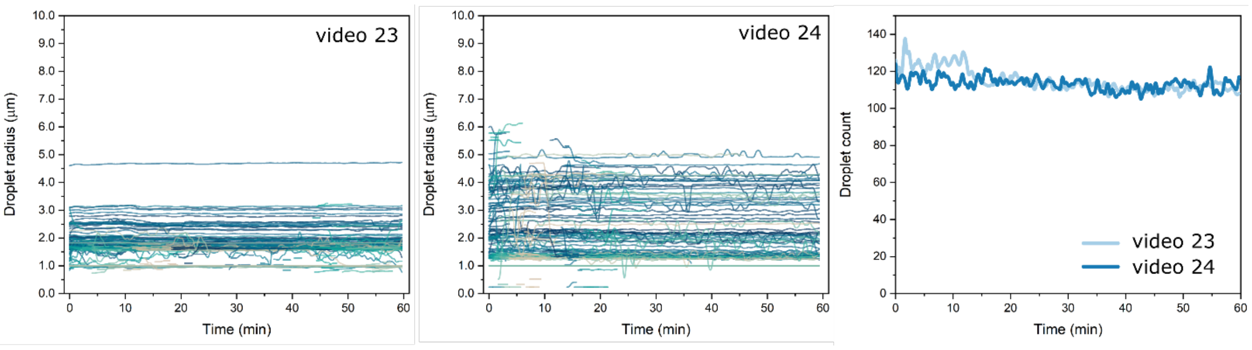

Supplementary figure 7. Radii traces and droplet count during passive coacervate experiments, at 1 mM (video 23) and 3 mM of ATP (video 24) and both at 20 uM of K_{72}. In the main text, video 23 is the low volume fraction example, while video 24 is the source of the high volume fraction traces. Notice the time scale is in minutes in these plots.

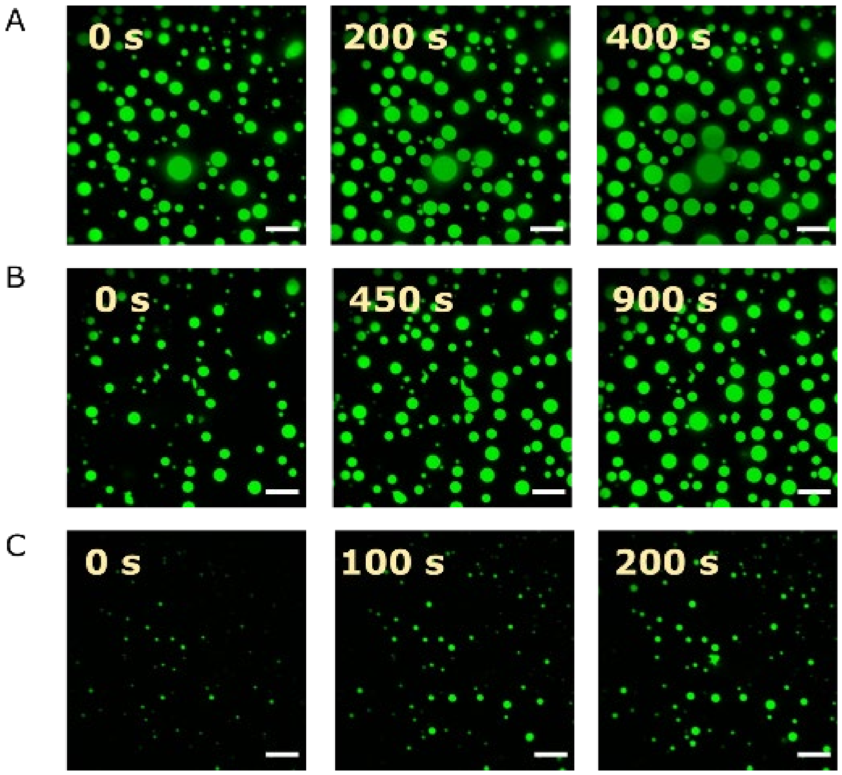

Supplementary figure 8. Expanded (full) frames of the images shown in main Figure 3. Scale bar: 10 µm.

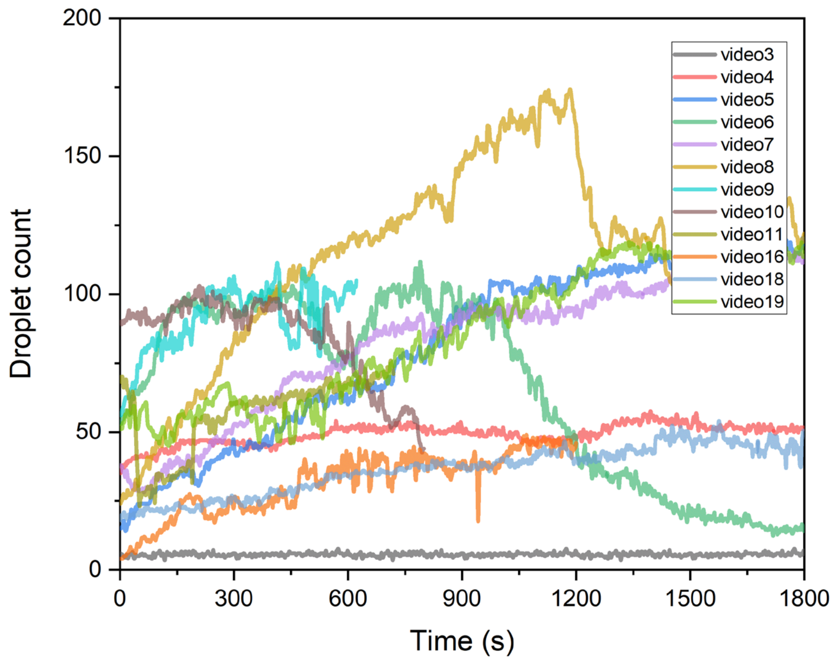

Supplementary figure 9. Droplet count during active droplet experiments. See Supplementary table 1 for the meaning of the short names.

III. Supplementary information to the section: Suppressed Ostwald ripening of complex coacervate droplets

We estimated the potential interference of coalescence and Ostwald ripening to reaction-driven growth with the following set of equations and parameters as in reference [1].

- Rate of change in average droplet volume (proportional to r^3) by Brownian motion-induced coalescence (BMC):[1]

$$\frac{\mathrm{d}\langle r^3\rangle}{\mathrm{d}t} = k_{\mathrm{BMC}} = \frac{2\theta k_{\mathrm{B}}T}{\pi\eta}$$

$$k_{\mathrm{BMC}} = 93\ \mu\mathrm{m}^3\mathrm{h}^{-1}$$

$$\Delta R(60\ \mathrm{min}) = 11\ \mu\mathrm{m}$$

- Rate of change in average droplet volume by Ostwald ripening (OR):[1]

$$\frac{\mathrm{d}\langle r^3\rangle}{\mathrm{d}t} = k_{\mathrm{OR}} = \frac{8\gamma D C_{\mathrm{sat}}(V_{\mathrm{m}})^2}{9R_g T}$$

$$k_{\mathrm{OR}} = 5 \text{ x } 10^{23}\ C_{\mathrm{sat}}\ell^5 = 28.0\ \mu\mathrm{m}^3\mathrm{h}^{-1} \quad (\mathrm{K}_{72})$$
$$= 1.8\ \mu\mathrm{m}^3\mathrm{h}^{-1} \quad (\mathrm{ATP})$$

$$\Delta R(60\ \mathrm{min}) = 3.0 - 7.4\ \mu\mathrm{m}$$

Supplementary table 2. Estimated typical properties of the coacervate droplets used.

Parameter	Description	Value	Source
$\boldsymbol{\theta}$	Volume fraction of coacervate phase	1-5%	centrifugation of coacervates, confocal slice extrapolation
$\boldsymbol{\eta}$	Viscosity of the medium	10^{-3} Pa · s	water
$\boldsymbol{\gamma}$	Coacervate surface tension	5.0×10^{-4} N m^{-1}	[2]
$\boldsymbol{D}$	Diffusivity in dilute phase	$\frac{k_B T}{6\pi\eta\ell}$	Stokes-Einstein (using molecular length scale ℓ as effective radius)
$\boldsymbol{C_{\mathrm{sat}}}$	Saturation concentration	5 µM (K_{72})<br>1 mM (ATP)	Minimal concentrations tested that led to coacervation (Figure 2B)
$\boldsymbol{\ell}$	Molecular length scale	2.5 nm (K_{72})	[3]
		0.77 nm (ATP)	[4]
$\boldsymbol{V_{\mathrm{m}}}$	Molecular volume	6.5×10^{-26} m^3 (K_{72})	$\frac{4}{3}\pi\ell^3$
		5.3×10^{-28} m^3 (ATP)	
$\boldsymbol{z}$	Net charge of K_{72} with GFP tag	+65	at pH 7.4
$\boldsymbol{\kappa}$	Inverse Debye length	7.1×10^8 m^{-1}	$\kappa \approx \sqrt{10\,I}$, for I = 50 mM salt
$\boldsymbol{\alpha_{\mathrm{E}}}$	Electrostatic penalty constant	1.7×10^{-35} m^2	see text
$\boldsymbol{\alpha_{\mathrm{OR}}}$	Ostwald ripening constant	6.5×10^{-29} m	see text

We do not observe shrinkage of complex coacervate droplets, either in active or passive systems. We propose that Ostwald ripening can be suppressed in complex coacervates by the electrostatic energy cost of removing a charged component from a small droplet (mechanism i), or by the energy barrier associated with removing an electroneutral complex in the form of a mini-droplet of coacervate phase from any droplet (mechanism ii).

In mechanism (i), the removal and transport of a point charge K_{72} (charge Q=+65e) from just outside droplet 1 (radius r_1, surface charge $-Q/4\pi r_1^2$) to just outside droplet 2 (radius r_2, without net surface charge) has the following energy difference:

$$\Delta U = (U_{E,2} + U_{C,2}) - (U_{E,1} + U_{C,1}) = \left[0 + \frac{2\gamma V_\mathrm{m}}{r_2}\right] - \left[-\frac{(ze)^2}{4\pi\varepsilon\kappa {r_1}^2} + \frac{2\gamma V_\mathrm{m}}{r_1}\right]$$

$$\Delta U = \frac{(ze)^2}{4\pi\varepsilon\kappa {r_1}^2} + 2\gamma V_\mathrm{m}\left(\frac{1}{r_2} - \frac{1}{r_1}\right)$$

$$\Delta U = \frac{\alpha_E}{{r_1}^2} + \alpha_\mathrm{OR}\left(\frac{1}{r_2} - \frac{1}{r_1}\right)$$

The free energy of the transfer is therefore positive for:

$$r_2 > \frac{{r_1}^2}{r_1 - \frac{\alpha_E}{\alpha_\mathrm{OR}}}$$

For typical values of α_{Ee} and α_OR for our system (estimated using the parameters in Supplementary table 2), we find a negative value for the critical r_2, i.e., the transfer is always endergonic regardless of the relative radii. The radius-dependency that usually drives Ostwald ripening is removed because to energy U_C (droplet potential due to increased Laplace pressure across the interface) it must be added an energy U_E (the potential created by charge separation), with opposite sign and stronger radius dependency (Supplementary Figure 10A). Moreover, if we include the fact that many protein condensates carry a small net surface charge,[5] the transfer likely becomes even more unfavourable, either because of additional electrostatic attraction at the source droplet, or a repulsion at the target droplet.

Alternatively, coarsening might happen by the transfer of electroneutral complexes of one or several K_{72} bound to ATP, which we view as mini-droplets of coacervate phase, from a small to a larger droplet (mechanism ii). However, this involves crossing an energy barrier formed by the loss of entropy of the K_{72} with associated ATP molecules, and arguably, creating the interface of the electroneutral complex. The entropy loss can be estimated from the ratio of the volume of the original droplet and the electroneutral complex. Assuming that the K_{72} and ATP molecules could freely move in the original droplet, which is supported by complete FRAP recovery (Supplementary figure 4), we estimate the entropy loss as follows:

$$-T\Delta S = -TS_\mathrm{complex} + TS_\mathrm{drop} = -\frac{kT}{2}\ln\left(\frac{V_\mathrm{complex}}{\ell^3}\right) + \frac{kT}{2}\ln\left(\frac{V_\mathrm{drop}}{\ell^3}\right) = \frac{kT}{2}\ln\left(\frac{V_\mathrm{drop}}{V_\mathrm{complex}}\right)$$

$$= kT\ln\left(\frac{4\pi r_1^3}{V_\mathrm{m,K72} + 17V_\mathrm{m,ATP}}\right)^{½}$$

where we have assumed the smallest possible transferable, electroneutral complex to consist of a single K_{72} and 17 associated ATP molecules. The factor 2 in the denominator corrects for the additional degrees of freedom of the complex and coacervate source droplet together.

The electroneutral complex can be regarded as a small droplet with an interfacial area A_{complex} and an interfacial energy:

$$U_S = \gamma A_{\text{complex}} = \gamma\,(36\pi)^{1/3}\left(V_{\text{m,K72}} + 17\,V_{\text{m,ATP}}\right)^{2/3}$$

We assume that the decrease in surface area of the droplet from which the electroneutral complex is removed is negligible, which is reasonable for droplets larger than several tens of nm. The total energy barrier is the sum of these two contributions (U_S - $T\Delta S$), which is prohibitively large (> 10 kT) for all droplets larger than 10 nm using the parameter estimates in Supplementary table 2. Both terms are of the same order of magnitude for these parameter values, and each will thus effectively suppress Ostwald ripening by slowing it down to a negligible rate.

We conclude that both a ripening mechanism based on transport of charged components and a mechanism based on transport of electroneutral complexes are hampered, one because the process is endergonic, the other because of a prohibitively large energy barrier. Overnight observations of passive complex coacervate droplets, in which we observe no change in size of any of the droplets are in agreement with this analysis (Supplementary figure 10b).

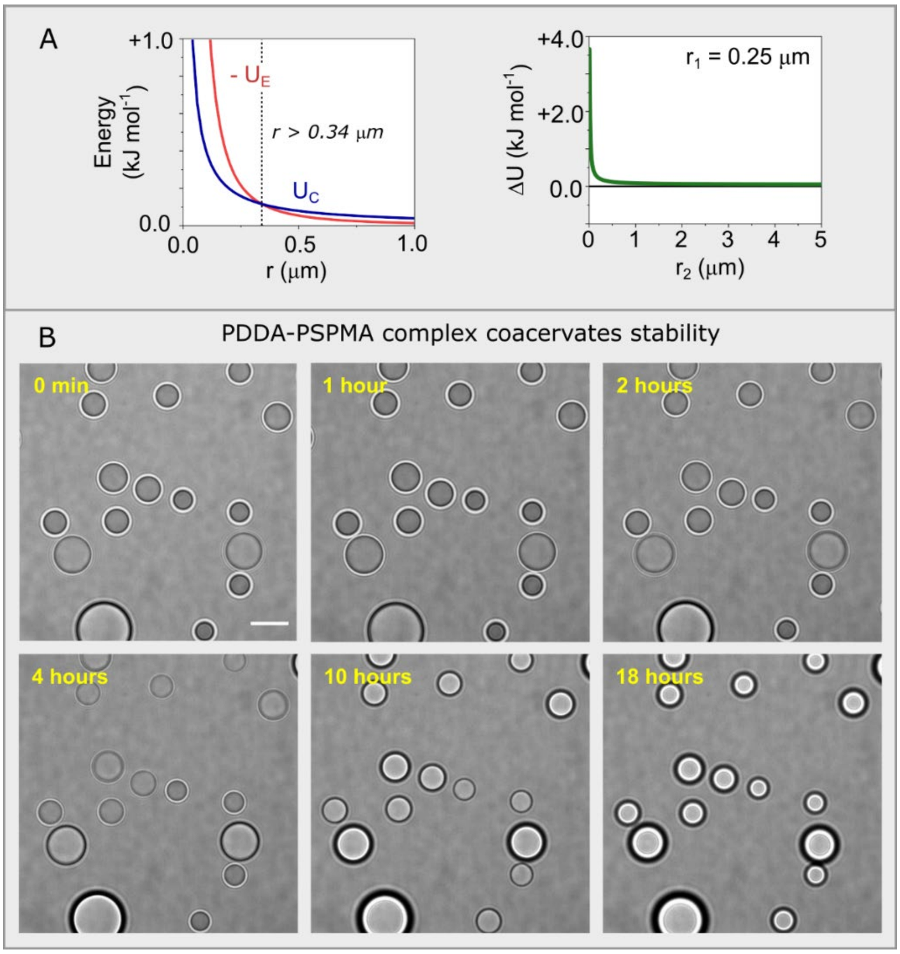

Supplementary figure 10. Estimates of energy associated with Ostwald ripening of complex coacervates and absence of coarsening in polymeric complex coacervates. (a) Additional plots for ripening mechanism (i), where a charged complex is removed and transported from a droplet. The left plot is a version of the plot in main Figure 4c, showing that beyond a droplet radius of 0.34 μm, the electric component, U_E, overcomes the Laplace pressure-driven component, U_C. The consequence of this proximity is seen on the plot to the right: the energy difference to transfer a charged molecule from a droplet of radius r_1 to a droplet of radius r_2 is not exergonic, and essentially the same for any given r_2. (b) Bright-field micrographs of PDDA and PSPMA coacervates. These are complex coacervates like ATP-K_{72}, and lack any active process that could explain their size stability over 18 hours. PDDA = poly(diallyl dimethylammonium chloride) 200-350 kDa, PSPMA = poly(3-sulfopropyl methacrylate) 30 kDa. Scale bar (0 min): 10 μm. This experiment was repeated 2 times with similar results.

We note that our theoretical analysis is based on a highly simplified and some ways flawed view of the complex coacervate droplets and the coarsening process, with many limitations. In particular, it is not always clear what the molecular configuration of polyelectrolytes that form complex coacervates is in the dilute phase that coexists with the droplets. It has been argued that long polyelectrolytes with a high charge density undergo pairing of electrostatic blobs into electroneutral 'soluble' complexes.[6,7] In that case, viewing such complexes as part of the coacervate phase and attributing an interfacial energy to them may not be realistic. However, in our case and in others, the phase-separating molecules are small (ATP) or have a low to moderate charge density (K_{72}). In addition, our solution contains added salt, which reduces the electrostatic interactions in the dilute phase compared to the salt-free solutions typically considered in theory.[6,7] Based on these considerations, the complex coacervate components are likely unpaired in the dilute phase. Moreover, even very long and highly charged polyelectrolytes that would form pairing in the dilute phase in theory, have a slight mismatch in length or distance between charges in practice, which means that it is unlikely that a small electroneutral complex could be removed without electrostatic penalty.

IV. Supplementary information to the section: Growth at a single-droplet level

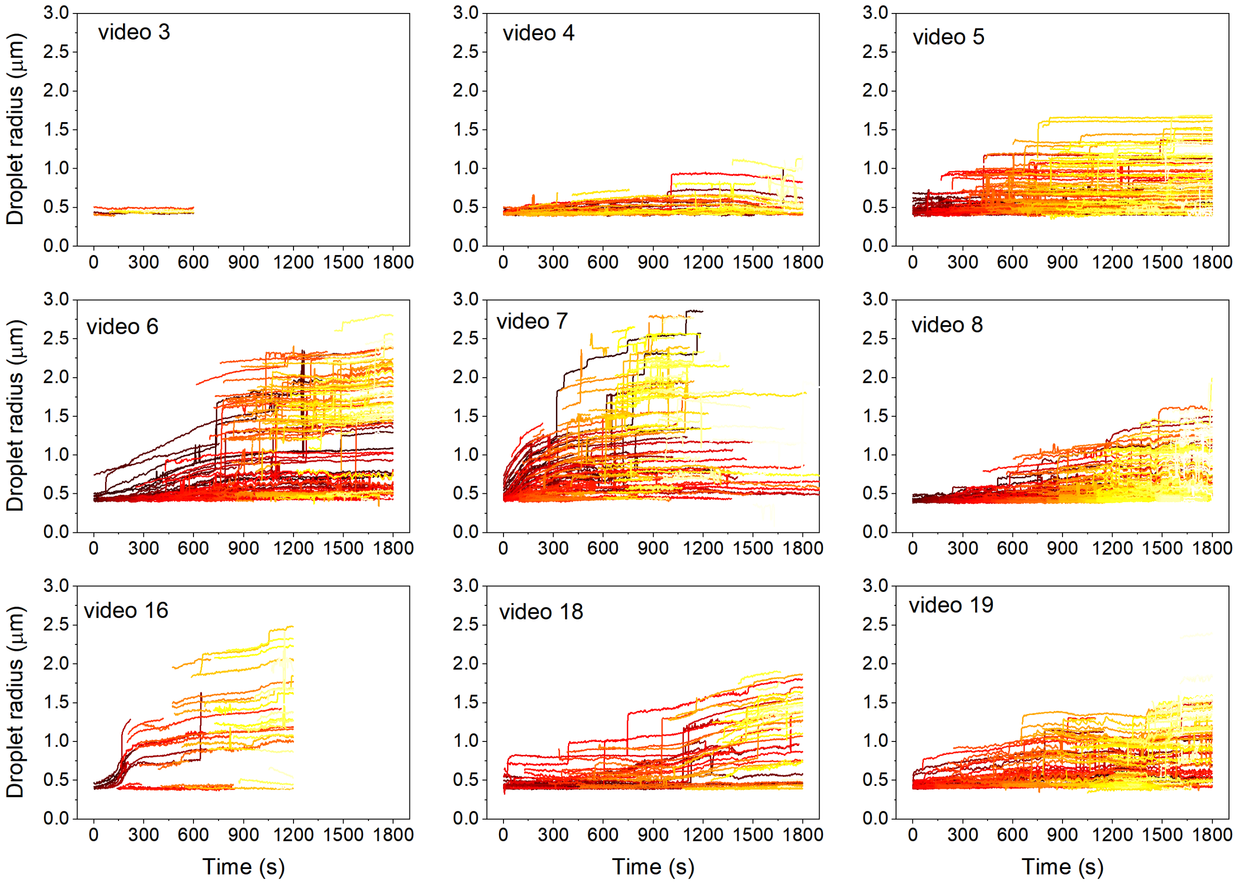

Supplementary figure 11. Radius traces during selected active droplet experiments. See Supplementary table 1 for the meaning of the short names. Video 6 shows the extended plot for the dataset in main Figure 5a.

Comparison between Michaelis-Menten estimation and volume trace profile

For our discussion of the enzymatic reaction, we use the following kinetic equations (typical Michaelis-Menten), where E stands for enzyme, S for substrate, and P for product.

$$E + S \rightleftarrows E - S \qquad k_1, k_{-1}$$
$$E - S \rightarrow E + P \qquad k_2$$
$$\frac{d[P]}{dt} = \frac{k_2[E]_0[S]}{\frac{k_{-1} + k_2}{k_1} + [S]}$$

To estimate ATP concentration profile over time, we consider ADP as substrate S, pyruvate kinase as E and ATP as the product P. We then use a first order approximation:

$$[S]_t = [S]_0(1 - e^{-k_{\text{obs}}t})$$
$$k_{\text{obs}} = \frac{k_2[E]_0}{\frac{k_{-1} + k_2}{k_1}} = \frac{v_{\max}}{K_{\text{M}}}$$

We can then obtain k_{obs} either by knowing $v_{\max}$ and K_{M} or directly from a kinetic monitoring of ATP concentration. We performed this monitoring in a previous publication, under similar conditions as the current study: 3 mM PEP, 3 mM ADP-Mg^{2+} and 50 mM HEPES buffer pH 7.4. The enzyme concentration in that case was 80 nM, but that can be accounted for as linear factor in k_{obs}. Supplementary figure 12a shows the reaction progress curve in a homogeneous solution, and Supplementary figure 12b shows the results in an emulsion (*i.e.,* the same conditions, but in presence of K_{72}). Because in the absence of K_{72} sample preparation before HPLC analysis is simpler, we were able to measure more time points and therefore this is the curve we fitted to obtain k_{obs}.

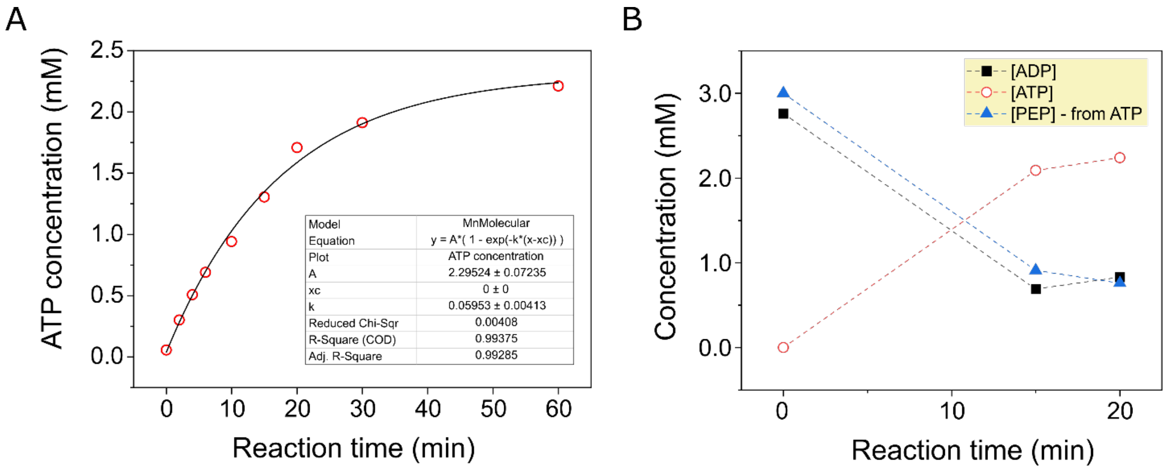

Supplementary figure 12. ATP formation kinetics in solution and in the presence of K_{72}. (a) Progress curve of the pyruvate kinase catalyzed-reaction in solution when $[ADP]_0 = [PEP]_0 = 3$ mM and enzyme concentration is 80 nM, fitted to obtain $k_{\text{obs}} = 0.09$ min^{-1}. (b) Formation of ATP in the presence of protein K_{72} (during coacervate formation). The total nucleotide concentration was measured with HPLC. Three different mixtures were prepared, and quenched with acetic acid at each time point. The emulsions contained: 20 µM K_{72}, 3 mM ADP, 3 mM PEP, 0.5 mM $MgCl_2$ and 0.42 µM PyK (exact same conditions as Video 6).

Supplementary table 3. Parameters used in the Michaelis-Menten (or solution phase) predictions of ATP concentration progress.

Pyruvate kinase concentration (nM)	k_{obs} (min^{-1})
80	0.059 *(fitted)*
140 *(as in video 18)*	0.30 *(calculated)*
420 *(as in video 6)*	0.10 *(calculated)*
$[\mathrm{ATP}]_t = [\mathrm{ADP}]_0(1 - e^{-k_{\mathrm{obs}}t})$	

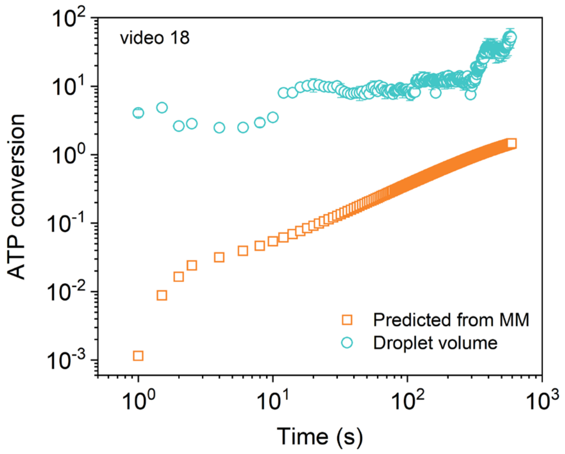

Supplementary figure 13. Log-log plot of ATP conversion as experimentally indicated by droplet growth (blue circles) and as predicted using $k_2 = 0.1$ min^{-1}.

V. Supplementary information to the section: Growth at a population level

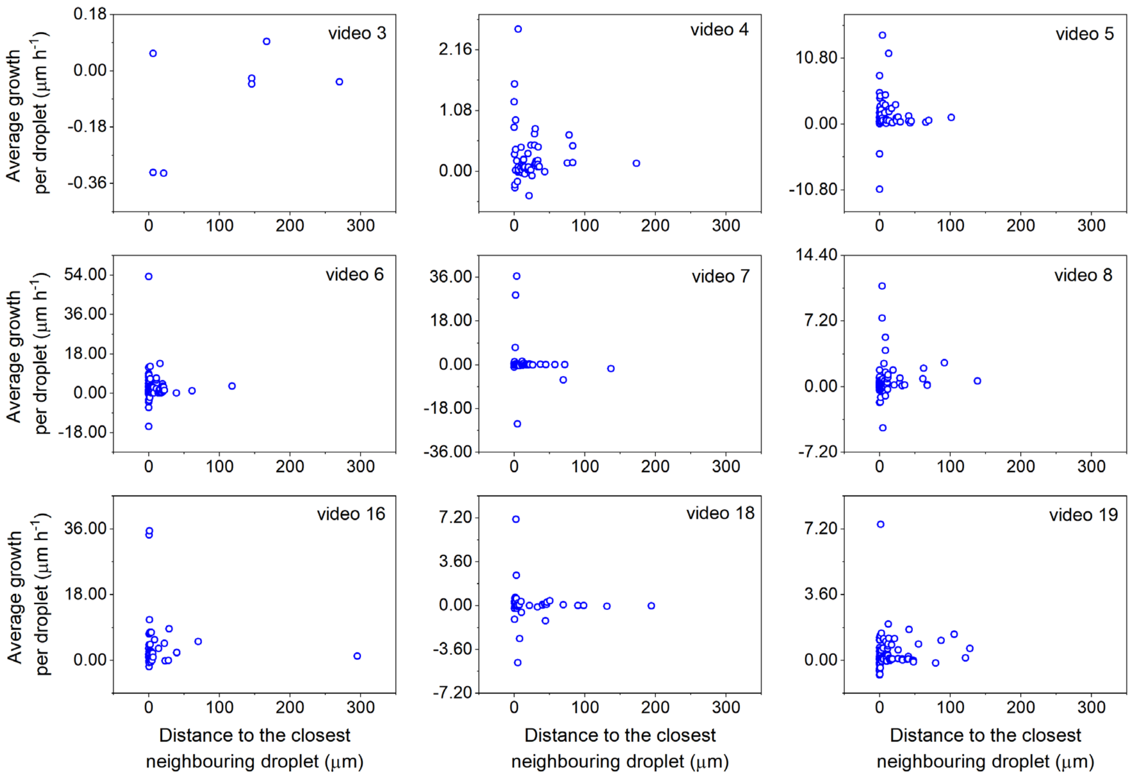

Supplementary figure 14. Relation between growth rate (averaged from the list of local growth rates) of a droplet and its position relative to other droplets. In other words, the relation between the growth rate of a droplet during an experiment and the presence of a nearby droplet. See Supplementary table 1 for the meaning of the short names.

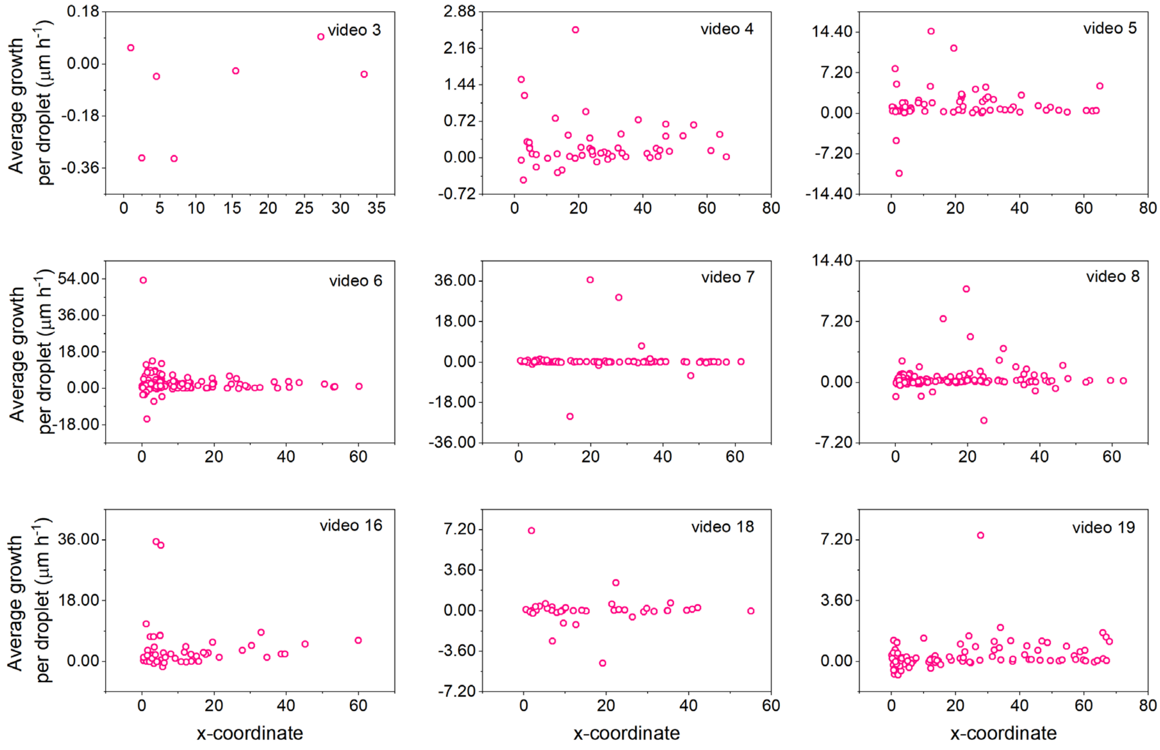

Supplementary figure 15. Relation between growth rate (averaged over the list of local growth rates) of a given droplet and its x-axis position in the well. See Supplementary table 1 for the meaning of the short names.

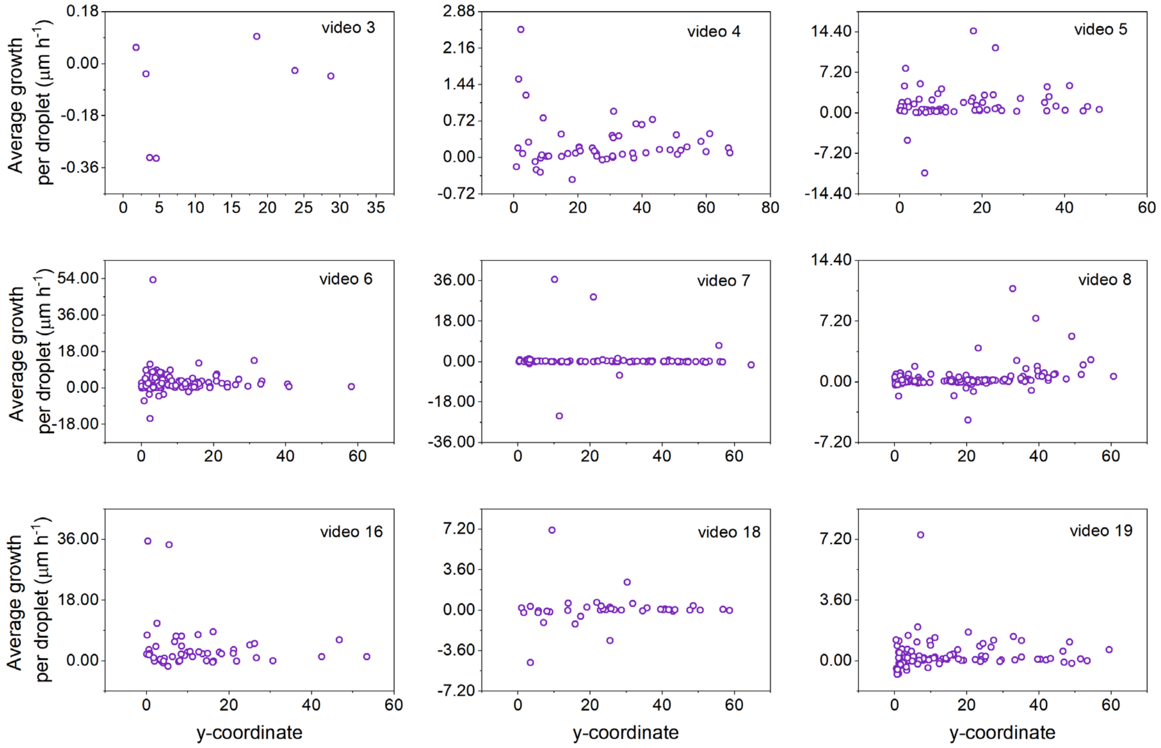

Supplementary figure 16. Relation between growth rate (averaged over the list of local growth rates) of a given droplet and its y-axis position in the well. See Supplementary table 1 for the meaning of the short names.

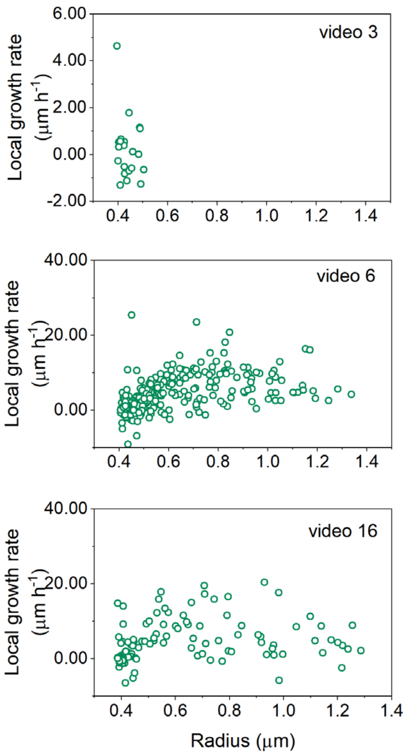

Supplementary figure 17. Relation between growth rate (taken from the list of all derivatives) and average droplet size at which the derivative was calculated. See Supplementary table 1 for the meaning of the short names.

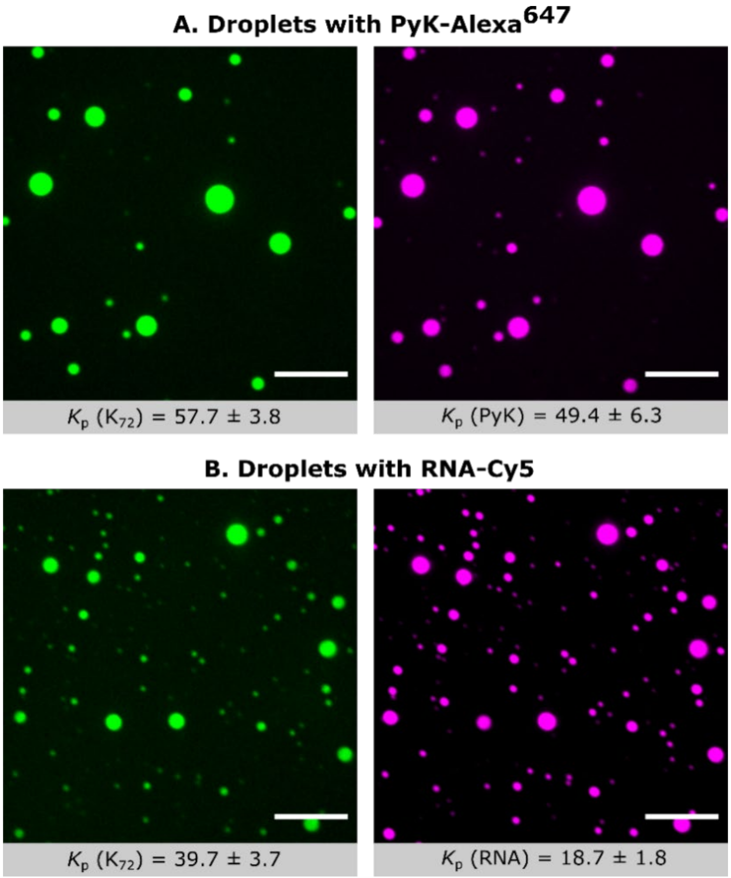

Supplementary figure 18. (a) ATP-RNA-K_{72} droplets containing Alexa-647 labelled PyK. (b) ATP-RNA-K_{72} droplets containing RNA-Cy5 (ss-$(ACGU)_6$-Cy5, 1 μM). RNA stands for ss-$(ACGU)_6$. For both panels: excitation channels are green (488 nm, GFP) and magenta (640 nm, Alexa-647); scale bars: 10 μm.

VI. Supplementary references

1. Berry, J. *et al.* RNA transcription modulates phase transition-driven nuclear body assembly - Supporting Information. *Proc. Natl. Acad. Sci. U. S. A.* 1–9 (2012).
2. Spruijt, E., Westphal, A. H., Borst, J. W., Cohen Stuart, M. A. & Van Der Gucht, J. Binodal compositions of polyelectrolyte complexes. *Macromolecules* **43**, 6476–6484 (2010).
3. Erickson, H. P. Size and shape of protein molecules at the nanometer level determined by sedimentation, gel filtration, and electron microscopy. *Biol. Proced. Online* **11**, (2009).
4. Rostovtseva, T. K. & Bezrukov, S. M. ATP transport through a single mitochondrial channel, VDAC, studied by current fluctuation analysis. *Biophys. J.* **74**, 2365–2373 (1998).
5. Welsh, T. J. *et al.* Single particle zeta-potential measurements reveal the role of electrostatics in protein condensate stability. *bioRxiv* 1–35 (2020) doi:10.1101/2020.04.20.047910.
6. De Gennes, P. G., Pincus, P., Velasco, R. M. & Brochard, F. Remarks on polyelectrolyte conformation. *J. Phys.* **37**, 1461–1473 (1976).
7. Delaney, K. T. & Fredrickson, G. H. Theory of polyelectrolyte complexation - Complex coacervates are self-coacervates. *J. Chem. Phys.* **146**, (2017).
