# Supplementary material for: Active coacervate droplets are protocells that grow and resist Ostwald ripening
Source: Nat Commun. 2021 Jun 21;12:3819. doi: 10.1038/s41467-021-24111-x (PMC8217494; doi:10.1038/s41467-021-24111-x)
Supplement: Supplementary file 2 — Description of Additional Supplementary Files [file 41467_2021_24111_MOESM2_ESM.docx]

**Description of Additional Supplementary Files**

**Title: Supplementary data 1.**

Description: Source data to the figures in the main article, including the videos files that were analysed. A description and details of the videos is included in the pdf file included in the dataset.

**Title: Supplementary data 2.**

Description: Confocal microscopy Z-stack data of a coacervate droplet, used for the 3D reconstruction shown in supplementary figure 5.
