# Supplementary material for: Active coacervate droplets are protocells that grow and resist Ostwald ripening
Source: Nat Commun. 2021 Jun 21;12:3819. doi: 10.1038/s41467-021-24111-x (PMC8217494; doi:10.1038/s41467-021-24111-x)
Supplement: Supplementary file 3 — Dataset 1 [file 41467_2021_24111_MOESM3_ESM.zip › Source data/Nakashima_Supp Video descriptions.pdf]

Supplementary videos file description

All files are the original raw data, with ImageJ's green or red LUT applied.

Filename	Scale	Frame rate	Speeding	Description
Video3.avi	141 nm/px	30 fps	60x	Active droplets
Video4.avi	141 nm/px	30 fps	60x	Active droplets
Video5.avi	141 nm/px	30 fps	60x	Active droplets
Video6.avi	141 nm/px	30 fps	60x	Active droplets
Video7.avi	141 nm/px	30 fps	60x	Active droplets
Video8.avi	141 nm/px	30 fps	60x	Active droplets
Video9.avi	141 nm/px	30 fps	60x	Active droplets
Video10.avi	141 nm/px	30 fps	60x	Active droplets
Video11.avi	141 nm/px	30 fps	60x	Active droplets
Video16.avi	141 nm/px	30 fps	60x	Active droplets
Video18.avi	141 nm/px	30 fps	60x	Active droplets
Video19.avi	141 nm/px	30 fps	60x	Active droplets
Video23.avi	400 nm/px	30 fps	30x	Passive coacervates
Video24.avi	400 nm/px	30 fps	30x	Passive coacervates
Video25.avi	1.88 µm/px	30 fps	150x	Passive oils
Video26.avi	1.14 µm/px	30 fps	300x	Passive oils
Video27.avi	141 nm/px	30 fps	60x	Active droplets
Video28.avi	141 nm/px	30 fps	60x	RNA-active droplets
Video29.avi	141 nm/px	30 fps	1800x	Passive coacervates
